# Pandemic and Partisan Polarisation: Voter Evaluation of UK Government Handling During Covid‐19

**DOI:** 10.1111/spsr.12457

**Published:** 2021-05-06

**Authors:** Tim Vlandas, Margaryta Klymak

**Affiliations:** ^1^ University of Oxford

**Keywords:** Partisanship, Political polarization, United Kingdom, Covid‐19, Government competence, YouGov survey

## Abstract

What is the effect of pandemics on partisan perceptions of government competence? Taking the case of Covid‐19 in the UK, we explore how voters’ assessments of the government’s handling of the economy and health were affected by four events: the first UK Covid‐19 death; the national lockdown; Boris Johnson’s hospitalisation; and Cummings’ scandal. Using a large representative weekly survey in the last year totalling over around 30’000 respondents, our results show that Labour voters had the worst assessments of government handling. The first death deteriorated perceptions of government handling of health among both Labour and Conservative voters, while Boris Johnson’s hospitalisation improved perceptions among most voters. Lockdown improved the perception of health handling but at the cost of more negative perceptions of its handling of the economy. The Cummings scandal had a negative effect on perceptions of government handling of economy but surprisingly improved perceptions of its handling of health.

## Zusammenfassung

Was ist der Effekt von Pandemien auf parteipolitische Wahrnehmungen der Leistungsfähigkeit einer Regierung? Am Fallbeispiel von Covid‐19 in Grossbritannien untersuchen wir, wie die Einschätzungen der Wähler zum Umgang der Regierung mit Wirtschaft und Gesundheit durch vier Ereignisse beeinflusst wurden: den ersten britischen Covid‐19‐Todesfall; den nationalen «Lockdown»; Boris Johnsons Krankenhausaufenthalt; und den Cummings‐Skandal. Anhand einer grossen repräsentativen wöchentlichen Umfrage im letzten Jahr mit insgesamt über 30’000 Befragten zeigt dieser Beitrag, dass Labour‐Wähler die Leistung der Regierung am schlechtesten einschätzten. Der erste Todesfall verschlechterte die Wahrnehmung der Regierungsleistung in Bezug auf Gesundheit bei Wählern sowohl von Labour als auch der Konservativen, während Boris Johnsons Krankenhausaufenthalt die Wahrnehmung bei den meisten Wählern verbesserte. Der Lockdown verbesserte die Wahrnehmung der Leistung bezüglich Gesundheit, jedoch auf Kosten einer negativeren Wahrnehmung des Umgangs mit der Wirtschaft. Der Cummings‐Skandal wirkte sich negativ auf die Wahrnehmung des Umgangs der Regierung mit der Wirtschaft aus, verbesserte jedoch überraschend die Wahrnehmung des Umgangs mit der Gesundheit.

## Résumé

Quels sont les effets des pandémies sur les perceptions partisanes de la performance gouvernementale? Cet article analyse comment quatre évènements liés au COVID‐19 au Royaume‐Uni ont affecté la façon dont les électeurs perçoivent la compétence gouvernementale: le premier mort du COVID‐19 au Royaume‐Uni, le confinement national, l’hospitalisation de Boris Johnson, et l’affaire Cummings. Nous utilisons un sondage à partir d'un échantillon de plus de 30 000 résidents britanniques. Nos résultats suggèrent que les électeurs du parti travailliste avaient la perception la plus négative du gouvernement conservateur. Le premier décès a détérioré la perception de la compétence gouvernementale dans le domaine de la santé, alors que l’hospitalisation de Johnson a amélioré la perception de la performance gouvernementale. Le confinement national a amélioré les perceptions de la compétence gouvernementale dans le domaine de la santé au détriment de celui de l’économie. Le scandale de Cummings a eu un effet négatif sur la perception de la compétence économique, mais – paradoxalement – positif pour la santé.

## Introduction

The recent Covid‐19 pandemic represents a unique opportunity to examine whether and how a shock to the distribution of economic and health risks affects differences in voters’ evaluations of government competence along partisan lines. Indeed, there is a large literature on political polarization^1^ and whether the partisan affiliation of voters affects their perceptions and evaluations of governments, their policies, their responsibility for bad outcomes, their competence and/or their performance.^2^ However, the question of whether and how pandemics affect the extent to which partisanship shapes perceptions of government performance has so far received limited attention. In particular, the effect of the Covid‐19 pandemic, the events surrounding it, and the policy responses that governments have adopted to react to it, are theoretically ambiguous and empirically untested.

To address this gap, we tackle two research questions of theoretical and policy relevance. First, what is the effect of Covid‐19 related policies, announcements and events on perceptions of government competence during the pandemic? Second, do these effects depend on the partisan affiliation of different groups of voters and do partisan differences remain once we control for other relevant socio‐demographic and economic covariates? Both questions are not *a priori* straightforward to theorise since the extent to which individuals update their political preferences and evaluations of governments in response to shocks is contested.

Our analytical starting point is that the recent Covid‐19 crisis and subsequent policies and events generate a series of shocks to the (perceived or real distribution of) economic and health risks that voters face. Since “voters’ evaluations and judgments are conditioned by their prior political beliefs, primarily their partisanship” (Tilley and Hobolt 2011: 318), we can expect these risks to feed into the evaluation of government competence along partisan lines. This partisan evaluation might occur because a voter might have ideological or identity‐based attachments to a party (e.g. Campbell et al. 1960; Green et al., 2020). But there could also be material reasons for these partisan differences, with right‐wing parties often representing older and richer voters, while left‐wing parties usually capture younger and/or low income voters.

To examine our two research questions, we use a large representative survey in the UK, collected every week between June 2019 and June 2020, which asks respondents about their approval of the government’s handling of the economy and the National Health Service. Our empirical approach relies on a series of linear probability regressions^3^ to test the effect of four events: the first UK Covid‐19 death; the declaration by the Conservative government of a national lockdown; the hospitalisation of Prime Minister Boris Johnson; and the scandal surrounding Dominic Cummings’ non‐compliance with lockdown regulations. We do not have clear expectations about the effects of these events on partisan polarisation. On the one hand, they could exacerbate partisan differences between individuals that are affected to varying extents by events or have different partisan alignments with the incumbent. On the other hand, when faced with significant existential and/or national threats, ideological differences between voters with distinct partisan leanings might fade.

We start by running regression models that investigate the effect of events and partisanship on the approval of the government’s handling of health and the economy. First, our results indicate a higher average evaluation of government handling of both economy and health among Conservatives, than among Labour and Scottish National Party (SNP) voters, with Liberal Democrats occupying an intermediate position. Second, different events had very distinct effects on each dimension of government handling: the first death was only negatively associated with the health dimension, while the Cummings scandal was only negatively associated with the economic dimension, and Boris Johnson’s hospitalisation improved assessments on both dimensions. The lockdown had opposite effects on each dimension: positive on the health dimension, but negative on the economic dimension, as one would expect. Third, we then interact our partisan variable with each event. This reveals that the first death had a negative impact on perceptions of government handling of health among both Labour and Conservative voters. Equally, Boris Johnson’s hospitalisation improved perceptions among most voters on both dimensions. By contrast, Conservative voters reported lower evaluations of government handling of economy after lockdown, whereas it was the opposite for health. Among Conservative voters, the Cummings’ scandal had a negative effect on perceptions of government handling of the economy, but this effect was positive for health.

This research note brings together the literature on partisan evaluations of government competence and the emerging debate about the political consequences of pandemics, and in particular, the effect of the pandemic and associated policy measures on political trust and government support (e.g. Bol et al. 2020; Deslatte 2020; Devine et al. 2020). Previous studies found that lockdowns increased satisfaction and trust in governments. While this state of the art is valuable and a useful starting point, we go beyond it in several ways. First, we distinguish between different dimensions of competence by using distinct measures of perceptions of handling of the economy and of the National Health System. Second, we are able to identify whether the evaluation of government handling depends on which political party the respondent voted for in the last election, thereby contributing to the literature on partisan evaluation of government competence. Third, we draw on a series of Covid‐19 related events that affect respondents to distinct extents and in different ways.

Taken together, our findings contribute to our understanding of views about government competence and political polarisation during pandemics. The mixed effects of the Cummings’ scandal might partially explain why Cummings was able to stay on. Similarly, our findings suggest that Johnson’s hospitalisation might have benefitted the government. The political cost‐benefit of the lockdown may also be appealing to a Conservative government intent on improving their image on health, while ‘owning’ the economic issue, thereby allowing them more margin of manoeuvre to sustain reputational damage. Overall, our results do not provide uniform and unambiguous evidence for an inevitable partisan polarization in the UK during the pandemic: while some initial assessments of government handling follow well‐established partisan lines, different events and policies have in some cases brought distinct group of voters closer in their perception of government competence. However, this depends crucially on both the nature of the event and the dimension of government competence that is being assessed.

The rest of this research note unfolds as follows. The first section describes our data and discusses our empirical approach. The next section presents our baseline results, while the last section concludes with some wider implications.

## Data and Empirical Strategy

The pandemic presents policy makers with a unique dilemma because it threatens the health of individuals, but restrictions on behaviour and activities that are designed to contain its spread might unwittingly harm the economy. Equally, voters are affected both economically and in their health, and might as a result alter their assessments of government handling on both dimensions. The extent to which these assessments evolve over time may in turn crucially depend on the occurrence of different events, the effectiveness of government policies, and individuals’ pre‐existing partisan leanings.

To explore these dynamics necessitates survey data that covers a large number of individuals over a long time period, before and after the pandemic as well as different events and policy announcements. The survey also needs to include information about individual perceptions of government handling, individuals’ voting behaviour, and relevant socio‐demographic and socio‐economic characteristics. The YouGov weekly tracker survey^4^ fulfils these requirements since it is a large representative weekly survey of UK respondents over a period of 12 months, totalling over 30’000 respondents. It also captures a wide range of background characteristics and, crucially for the purpose of creating our dependent variables, asks respondents how they think the government is handling the economy and NHS. Both our dependent variables are dummy variables; coded one if a respondent thinks the government is performing well on the economy and the NHS respectively, and zero otherwise.

We run a series of linear probability models that examine the effects of four exogenous events. Our empirical approach aims to estimate the probability of approving the governments’ handling and our specification is as follows:

$$\begin{array}{cc} y_{i,r,t}= & \alpha +\beta {\text{Event}}_{i,t}+\gamma {\text{Pastvote}}_{i,t}+\delta {\text{Demographic}}_{i,t}+\eta \text{Socio}‐{\text{economic}}_{i,t}\\ & +\varphi \text{Covid}\;{\text{Death}}_{t}+\theta_{r}+\text{Trend}+e_{i,r,t}; \end{array}$$

Our outcome y_irt_ captures our two dependent variables: whether an individual *i* living in region *r* at time *t* (i.e. time at which respondents are surveyed) thinks that the government is handling (1) the National Health Service and (2) the economy well. The main regressors of interests are past vote and events. Demographic controls include four age groups, an indicator of whether a respondent is single or in a relationship, and a respondent’s gender. Socio‐economic controls include variables capturing labour market status, income group and educational levels (for more details about our variables, please see table A1 in the appendix). Using data from the UK Office of National Statistics, we control for the temporal variation in the incidence of Covid‐19 spread captured by the number of Covid‐19 related death in the UK prior to the interview of the respondent.^5^ We also include a time trend and region fixed effects. Heteroskedasticity‐robust standard errors are clustered at the day and region levels.

The variable ‘past vote’ is based on how respondents voted in the 2019 General Election, while the variable ‘event’ takes the value one if the individual was surveyed after a particular event, and zero otherwise.^6^ More specifically, we focus on four events that have taken place in the period during which our survey was fielded. Building on previous studies, we expect these events to affect perceptions of (and trust in) government competence (cf. Bhatti et al. 2013; Bowler and Karp 2004; Dancey 2012; Maier 2011).

The first event is the news of the first Covid‐19 death in the UK, which occurred on 5^th^ March 2020 and captures the beginning of the pandemic in the UK. The second event is the announcement of national lockdown on 26^th^ March 2020, which captures the main policy response with wide‐ranging effects on people. The likely impact of these first two events on assessments of government competence are not theoretically clear: recent studies indicate lockdown increased support for the incumbent and trust in the government (e.g. Bol et al. 2020) but have not specifically focused on partisan dynamics or on other events that we consider here.

The third and fourth events capture major scandals/events in government. The third event is the admission of British Prime Minister Boris Johnson to hospital on 5^th^ April 2020 after testing positive for Covid‐19. The fourth event is the scandal surrounding Dominic Cummings’ non‐compliance with lockdown regulations on 22^nd^ May 2020. As can be seen in Figure B1 in the appendix, the third and fourth events spurred a lot of interest in Google searches. We see the Cummings and Boris Johnson’s events as good cases to focus on because they might affect voters’ perception of the justification and rationale for government handling of the pandemic, hence one could expect they would affect the perceptions of government competence.

The potential effects of the third and fourth event are not a priori clear. For Johnson’s hospitalisation, we could reasonably expect two competing effects. On the one hand, it could elicit sympathy for his predicament. On the other hand, it could suggest a lack of seriousness and possibly limited compliance with government rules to prevent the spread of the virus. We could further expect the sympathy mechanisms to be stronger for the policy domain, which is closer to the issue, i.e. health. Equally, for the partisan mediation of this effect, one could posit that the sympathy effect would dominate for his supporters (i.e. Conservative voters), while the lack of seriousness effect would dominate among his detractors (i.e. Labour voters). With respect to the Cummings scandal, we would expect this event to reduce the perception of government competence, because it shows that public officials do not follow the government’s rules, but whether and how this may depend on the partisan affiliations of voters is partly indeterminate.

How are perceptions of government handling shaped by the interaction between which parties individuals voted for and the occurrence of a particular event? This question echoes a wider literature about whether the partisan affiliation of voters affects their perceptions and evaluations of governments, their policies, their responsibility for bad outcomes, their competence and/or of their performance (e.g. Anderson et al. 2004; Campbell et al. 1960; Evans and Andersen 2006; Johnston et al. 2005; Lewis‐Beck et al. 2008; Redlawsk 2002; Tilley et al. 2008; Tilley and Hobolt 2011; Wlezien et al. 1997). Given this focus, it is important to check that the events do not affect the sampling of individuals who voted for different political parties. Table B7 in the appendix presents the summary statistics by party voted before and after each event. We find no evidence for a statistically significant difference in voters sampled before and after each event.

The summary statistics before and after the four events for all variables are shown in Tables B1 to B4 in the appendix. In terms of partisan differences, there are substantial differences between Labour and Conservative voters in their assessment of government handling (appendix table B5). It is worth briefly comparing the responses to our two dependent variables before and after each event. Health handling appears to be significantly lower after the first death in the UK. By contrast, respondents’ support for governments’ handling of both economic issues and National Health Service have increased after the lockdown, which is in line with previous literature suggesting that trust in government increased after the announcement of the lockdown (e.g. Bol. at al. 2020). After the lockdown, the perception of the handling of health increased clearly for both parties and the perceptions of the government’s handling of the economy for Labour voters increased moderately (appendix table B6). Mean values for government handling of health and economy also appear higher and statistically significant after Boris Johnson’s hospitalisation.

## Results

In this section, we present the results from a series of OLS regressions to evaluate the extent to which the four events and partisan differences change perceptions of government handling of economy and health during pandemic times. In a second step, we seek to assess whether these partisan differences are themselves exacerbated or reduced by these events. Thus, we add an interaction term between past vote and event, and report the average marginal effect of each event conditional on partisanship.

**Figure 1:**
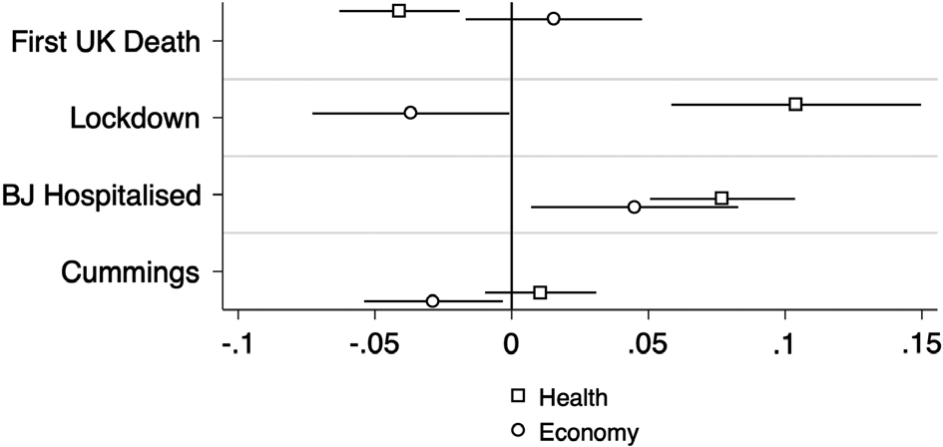
Effect of four events on perceptions of government handling of health and economy *Note:* The figure plots the main results of eight distinct regressions. Each coefficient is represented by a square or circle, while the bars indicate the 95% confidence intervals. The coefficients capture the effect of four independent variables (for the four separate events), on two dependent variables (perceptions of government handling of health and economy, respectively). All regressions include control variables as discussed in the Data and Empirical Strategy section.

First, we discuss the effect of the four events on our two dependent variables^7^. Figure 1 shows that the first UK death is negatively associated with the government’s handling of health, but has no statistically significant association with its handling of the economy. Next, the announcement of a national lockdown is negatively correlated with reporting that the government handles the economy well, but positively correlated with the handling of health. This points to the possibility that the national lockdown antagonised the parts of the Conservative electorate who care more about the economy, such as relatively well‐off middle aged supporters, while increasing support among those whose primary concerns lie with the health system, most notably the ‘grey voters’ but also traditionally Labour voters^8^. Johnson’s hospitalisation improved perceptions of the government’s handling of both economy and health, consistent with a sympathy mechanism. By contrast, the Cummings scandal seems to have reduced perceived competence on the economy, consistent with the notion that it signalled to the population that its public officials do not follow their own rules. However, it surprisingly did not have a statistically significant effect on health, presumably because it happened quite some time after the lockdown began, the economic costs of which could be justified more easily when government officials are seen to comply with their own rules.

**Figure 2:**
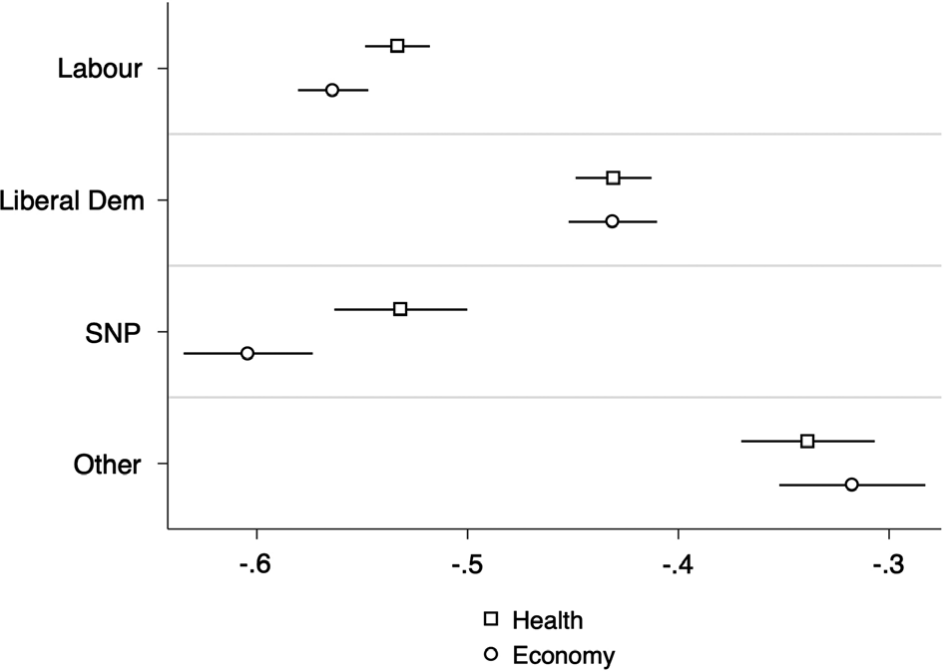
Effect of partisanship on perceptions of government handling of health and the economy *Note:* The figure plots the main results of eight distinct regressions. Each coefficient is represented by a square or circle, while the bars indicate the 95% confidence intervals. The coefficients capture the effect of partisanship (voting for Labour, Liberal Democrat, or SNP – relative to the Conservative baseline), on two dependent variables (perceptions of government handling of health and economy, respectively). All regressions include control variables as discussed in the Data and Empirical Strategy section.

Next, we present the differences between voters in Figure 2, which plots the effect of being a Labour, Liberal Democrat, SNP or 'Other party' voter relative to a baseline (which is set as Conservative voters) on individuals’ assessments of government handling of economy and health between June 2019 and June 2020. Voters of the Labour, Liberal and SNP parties are all less likely to think that the government is handling the NHS and the economy well, suggesting that assessments of government during a pandemic remain strongly partisan. The negative effect is especially strong for SNP voters^9^ and the economy, while for the Liberals it less marked although the magnitude of the effect is still substantial. Crucially, these effects endure despite controlling for relevant covariates such as income, education, age, gender, labour market and marital status. The fall in the size of the coefficients for the party vote as one introduces the relevant covariates is relatively mild (see Tables C1 to C2 in the appendix).

It is worth noting that consistent with a partisan story, the elderly and higher incomes have more positive assessment of the handling of the economy, while the unemployed, those in the North and Midlands, and the highly educated have more negative evaluations (appendix Table C1). By contrast, there is no age (or retirement) effect for health concerns, once work status, income and education levels are taken into account. Women, despite their lower fatality rates, and education are negatively associated with evaluations of government competence on health (appendix Table C2). Covid‐19 deaths are positively correlated with handling of both the economy and health.

**Figure 3:**
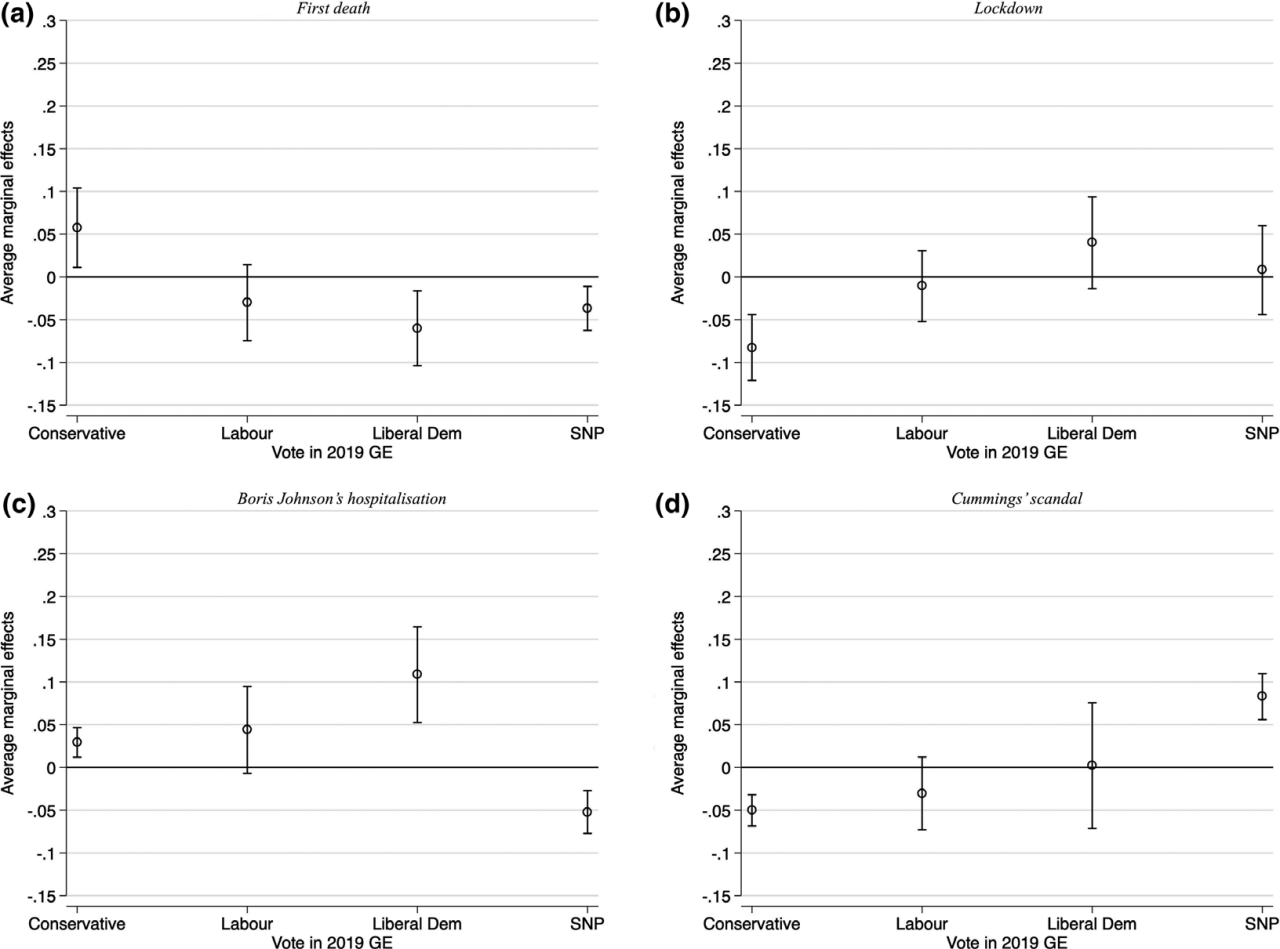
Effect of events on government handling of economy, conditional on partisanship *Note:* These figures plot the average marginal effects (with 95% confidence intervals) of each event, conditional on partisan affiliation, on the dependent variable (government handling of economy). The “Other” category was omitted from the graphs but was included in the regression specification.

In Figure 3, we turn our attention to whether the effects of our four events on perceptions of government handling of the economy are conditional on partisanship as captured by past vote (see appendix C for full results)^10^. We present the results for a full model with all controls but also show results with a baseline specification without controls in the appendix. The effect of the first death on government handling is negative and statistically significant for Liberal Democrats and SNP voters, but positive for Conservative voters, and statistically insignificant for Labour. The magnitudes of the effects are not particularly large. By contrast, the lockdown only had a negative effect on perceptions of handling of economy among Conservative voters^11^. Next, the hospitalisation of Boris Johnson changed perceptions of government competence positively for most parties. Interestingly, this effect was strongest among Liberals, followed by Labour and then Conservative voters, although the confidence intervals in the first two suggest imprecise estimates, whereas SNP voters lowered their perception of competence as a result. Finally, the Cummings scandal led to more negative perceptions of government handling of economy among Conservative voters.

**Figure 4:**
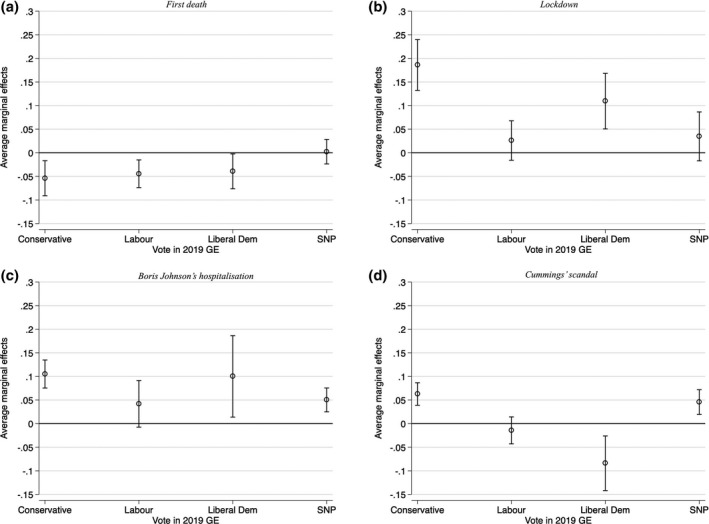
Effect of events on government handling of health, conditional on partisanship *Note:* These figures plot the average marginal effects (with 95% confidence intervals) of each event, conditional on partisan affiliation, on the dependent variable (government handling of health). The “Other” category was omitted from the graphs but was included in the regression specification.

The average marginal effects of the four events on changing perceptions of government handling of health conditional on partisanship are shown in Figure 4. The effect of the first death was statistically significant and negative (although not large in magnitude) for all main parties except SNP where the effect was not statistically significant. National lockdown had a large – equivalent to more than one third of the standard deviation in the dependent variable ‐ and statistically significant effect on positive perceptions of government handling of health among Conservative voters. The effect was also substantial for Liberal Democrats voters, but not statistically significant for other voters. Boris Johnson’s hospitalisation increased support for health handling among all parties except for Labour. Finally, the Cummings scandal increased positive assessment for health among Conservative voters, in contrast to its effect on economic handling.

Finally, we have estimated predicted values for each dependent variable for all four events for our four main groups of voters. The full results are shown in tables D2 and D3 in the appendix, and for reasons of space we can only summarise them here briefly in table 1 below. Overall, confirming our analysis of marginal effects, we can observe a complex picture of changing partisan evaluation of government competence in the domain of economy and health during the pandemic. When it came to their assessment of government handling of the NHS, the four events push Labour and Conservative voters in the same direction: both groups of voters exhibited higher predicted support after lockdown, Johnson’s hospitalisation and the Cummings Scandal; but lower predicted support after the first deaths. Despite a broadly similar direction of effects, the four events nevertheless tended to increase the gap between voters in their assessment of government handling in health, both between Labour and Conservative voters, and more generally in terms of the standard deviation in predicted position of all voter groups. Thus, partisan differences in perceptions of government competence in health has become more polarised as a result of the four events.

**Table 1:** Polarisation captured predicted probabilities of positive evaluations of government on health and economy

Dimension (right hand side) Event (below)	Economy		Health	
	Standard deviation	Change in predicted values (sign for Labour vs sign for Conservative)	Standard deviation	Change in predicted values (sign for Labour vs sign Conservative)
First death	Higher	Different ( + vs ‐)	Higher	Same (‐ vs ‐)
Lockdown	Lower	Same (‐ vs ‐)	Higher	Same (+ vs +)
Johnson Hospitalisation	Higher	Same (+ vs +)	Higher	Same (+ vs +)
Cummings	Lower	Same (‐ vs ‐)	Higher	Same (+ vs +)

Note:

These results are summarised based on tables D2 and D3 in appendix. The standard deviation captures the extent of variation in the predicted probabilities of positive evaluations of government handling for each voter group before and after each event. We also report whether the predicted perception has gone up (+) or down (‐) and whether it was in the same or a different direction for Labour versus Conservative voters.

The picture for government competence in the economic domain is less clear cut. For three out of the four events, Labour and Conservative voters moved in the same direction: the Lockdown and Cummings events reduced their perception of competence, whereas Johnson’s hospitalisation increased positive assessments. Yet, in the case of the hospitalisation the differences across the resulting position across all parties still increased, while the first death pushed Conservative and Labour voters in opposite directions, thereby also increasing partisan polarization.

## Conclusion

This research note has explored the effects of Covid‐19 events and policy measures on public perceptions of the UK government’s handling of the economy and National Health Service, paying particular attention to whether these events have increased or decreased differences in the perceptions of different voter groups. Using a large representative uninterrupted weekly survey in the UK, we have shown that Labour and SNP voters had the most sceptical assessments of government handling. Four distinct events then changed the relative assessment of different voters, depending on both the nature of the event and the dimension of competence that is at stake.

Indeed, the first death had a negative effect on the perceptions of government handling of health among both Labour and Conservative voters, while Boris Johnson’s hospitalisation improved perception among most voters for both dimensions. Lockdown improved perception of health handling but at the cost of lower perceptions of handling of economy among Conservative voters. It further led to a convergence in views about government handling of the economy towards a lower level among both Conservatives and Labour voters, whereas it led to partisan divergence in perceptions of its handling of health, despite leading to more positive views for all voter groups. The Cummings scandal had a negative effect on perceptions of government handling of economy among Conservative voters but – oddly – it appears to have raised their perception of health handling.

Our research note contributes to the emerging literature on the political consequences of pandemics and whether they lead to more polarisation in several ways. First, we offer an empirical analysis of a new country case study that has been particularly affected by Covid‐19, which can be contrasted with the results in other countries and/or outcomes: for instance, well‐being (Recchi et al. 2020) or compliance (Brouard et al. 2020) in France; the determinants of social distancing in the USA (Pedersen and Favero 2020), or the attitudes and beliefs of individuals towards Covid‐19 in Australia (Seale et al. 2020).

Second, we provide some of the necessary micro‐foundations about individuals’ views and preferences to help make sense of the policy responses (e.g. Di Mascio et al. 2020; Pierre 2020; Yan et al. 2020). We show that various events inside and outside the government’s control, such as the first Covid‐19 death or the health of the Prime Minister, can play a role in changing individuals’ perceptions of government competence. Thus, governments need to be careful about announcements and news as well as their potential effects on perceptions of competence and trust in government, which in turn may affect social stability, compliance, and ultimately the effectiveness of government responses during pandemics.

Finally, we document important partisan heterogeneity in the effects of different events on voters' evaluations of government action, which tie in to debates on whether the partisan affiliation of voters affects their perceptions and evaluations of governments, their policies, their responsibility for bad outcomes, their competence and/or their performance (e.g. Anderson et al. 2004; Campbell et al. 1960; Evans and Andersen 2006; Johnston et al. 2005; Lewis‐Beck et al. 2008; Redlawsk 2002; Tilley et al. 2008; Tilley and Hobolt 2011; Wlezien et al. 1997). In some cases, events are linked to more positive perceptions of competence across all groups of voters, which increases average support, but without altering existing gaps in the views of different voters. In other cases, we observe convergence resulting from individuals who have voted for the (Conservative) incumbent government lowering their perception of government competence, whereas other voters might develop more positive perceptions. In yet other instances, the events lead to increased divergence in views and hence further political polarisation in the views of different voters about government competence. The patterns are also contingent on policy dimension heterogeneity, most notably in the case of lockdown, which improved perceptions of government handling of the NHS, but lowered it on the economy.

Overall, pandemics appear to lead to a complex restructuring of partisan differences in the assessment of distinct voter groups of government competence. Although Lockdown, Johnson’s hospitalisation and the Cummings scandal have all tended to lead to more positive perceptions of government handling of health, the overall gap in perceptions between different groups of voters has widened, whereas the first death has tended to reduce perceptions of competence in health, but led to convergence in views across voters. By contrast, the Lockdown and Cummings scandal both led to more negative perceptions of competence in the economic domain, but also to a convergence of views across voters. Thus, the evolution of perceptions of government competence during a pandemic depends crucially on partisan affiliation, the policy dimension that is being considered as well as the nature and number of related events and policy responses.

## Supporting information

Supplementary Material

## Data Availability

While the disaggregated data is proprietary and owned by YouGov, the aggregated data of this study are openly available in YouGov at https://yougov.co.uk/topics/economy/trackers.

## Footnotes

^1^
E.g. Hetherington (2009), Lindqvist and Ostling (2010), Han (2015), Lindell et al. (2017), Ford and Jennings (2020).

^2^
E.g. Anderson et al. (2004), Campbell et al. (1960), Evans and Andersen (2006), Johnston et al. (2005), Lewis‐Beck et al. (2008), Redlawsk (2002), Tilley et al. (2008), Tilley and Hobolt (2011), Wlezien et al. (1997).

^3^
Note that using logistic regressions do not change our results.

^4^
For more information, please see: https://yougov.co.uk/topics/economy/trackers. We are grateful to Jonathan Van Parys and Emily Sane for their help in accessing and using this excellent proprietary survey dataset.

^5^
The results are robust to different thresholds, controlling for total death in the region to date and using Covid‐19 cases reported. We have also rerun our analyses while replacing deaths by hospitalisation data.

^6^
The Lockdown variable equals one in all weeks after the national lockdown announcement and zero otherwise. For the remaining three events, an event dummy takes the value of one in the week when the event is announced and zero in all other weeks.

^7^
We reproduce our results of the effect of events on perceptions of government handling of health and economy for each region (appendix figure D2).

^8^
See figure B2 in the appendix.

^9^
We rerun this analysis for Scotland (table D1 in appendix).

^10^
We have reproduced our analysis using logistic regression analysis instead of linear probability models (appendix D) and our findings are very similar.

^11^
Thus, in this case, ideology might be more important than being a supporter of the incumbent. Indeed, results in figure B2 in appendix suggest that Conservative voters value economy more and Labour health more.
