# Supplementary material for: Pandemic and Partisan Polarisation: Voter Evaluation of UK Government Handling During Covid‐19
Source: Schweiz Z Polit. 2021 May 6;27(2):325–38. doi: 10.1111/spsr.12457 (PMC8242772; doi:10.1111/spsr.12457)
Supplement: Supplementary file 1 — Supplementary Material [file SPSR-27-325-s001.docx]

**Appendix**

**Pandemic and Partisan Polarisation: Voter Evaluation of UK Government Handling During Covid-19**

**Margaryta Klymak (margaryta.klymak@some.ox.ac.uk) and Tim Vlandas (tim.vlandas@spi.ox.ac.uk)**

**Published in Swiss Political Science Review – accepted version March 2021**

**Contents**

[Appendix A: Description of data 3](#_Toc67592138)

[Table A1: variables description 3](#_Toc67592139)

[Appendix B: Summary statistics 4](#_Toc67592140)

[Table B1: Summary statistics before and after lockdown 4](#_Toc67592141)

[Table B2: Summary statistics before and after first UK Covid-19 death 5](#_Toc67592142)

[Table B3: Summary statistics before and after Boris Johnson’s hospitalization 6](#_Toc67592143)

[Table B4: Summary statistics before and after Dominic Cumming's non-compliance with lockdown regulations 7](#_Toc67592144)

[Table B5: Mean and standard deviation of answers to questions about handling of economy and health by labour versus conservative voters 8](#_Toc67592145)

[Table B6: Monthly average of answers to questions about handling of economy and health by labour versus conservative voters 8](#_Toc67592146)

[Table B7. Summary statistics of individuals with different last vote, before and after each event 9](#_Toc67592147)

[Figure B1: Google trends for search terms Cummings and Johnson in 2020 10](#_Toc67592148)

[Figure B2. Most important issues for conservative excluding labour voters 10](#_Toc67592149)

[Appendix C: Full results 11](#_Toc67592150)

[Table C1: Full results for government handling of economy 11](#_Toc67592151)

[Table C1 (cont.): Full results for government handling of economy 12](#_Toc67592152)

[Table C1 (cont.): Full results for government handling of economy 13](#_Toc67592153)

[Table C2: Full results for government handling of health 14](#_Toc67592154)

[Table C2 (cont.): Full results for government handling of health 15](#_Toc67592155)

[Table C2 (cont.): Full results for government handling of health 16](#_Toc67592156)

[Appendix D. Robustness checks. 17](#_Toc67592157)

[Additional tests 17](#_Toc67592158)

[Figure D1: Impact of events on perceptions of government handling of health and economy 17](#_Toc67592159)

[Figure D2: Impact of events on perceptions of government handling of health and economy for Scotland and England. 18](#_Toc67592160)

[Figure D3: Impact of partisanship on perceptions of government handling of health and the economy 19](#_Toc67592161)

[Figure D4: Average marginal effect of events on government handling of economy, conditional on partisanship, and including basic and full models 20](#_Toc67592162)

[Figure D5: Average marginal effect of events on government handling of health, conditional on partisanship, and including basic and full models 21](#_Toc67592163)

[Figure D6: Average marginal effect of events on government handling of economy, conditional on partisanship. Logistic models. 22](#_Toc67592164)

[Figure D7: Average marginal effect of events on government handling of health, conditional on partisanship. Logistic models. 23](#_Toc67592165)

[Figure D8: Average marginal effect of Cummings’ scandal on government handling of economy, conditional on partisanship and excluding June. 24](#_Toc67592166)

[Figure D9: Average marginal effect of Cummings’ scandal on government handling of health, conditional on partisanship and excluding June. 24](#_Toc67592167)

[Figure D10: Average marginal effect of Cummings’ scandal on government handling of economy, conditional on partisanship and controlling for hospitalization. 25](#_Toc67592168)

[Figure D11: Average marginal effect of Cummings’ scandal on government handling of health, conditional on partisanship and controlling for hospitalization. 25](#_Toc67592169)

[Figure D12: Average marginal effect of events on government handling of economy, conditional on partisanship and without controlling for Covid-19 related death 26](#_Toc67592170)

[Figure D13: Average marginal effect of events on government handling of health, conditional on partisanship and without controlling for Covid-19 related death 27](#_Toc67592171)

[Table D1: Marginal effects of events on government handling of economy, conditional on partisanship for SNP voters 28](#_Toc67592172)

[Predictive marginal effects for economy. 29](#_Toc67592173)

[Table D2. Predictive probability of respondent stating that government is handling the economy well. 29](#_Toc67592174)

[Table D3. Predictive probability of respondent stating that government is handling the health well. 30](#_Toc67592175)

# Appendix A: Description of data

### Table A1: variables description

| ***Variable*** | **Description** |
| --- | --- |
| *Economy Handling* | How well or badly do you think the government is handling the following issues? “The economy”. A dummy variable coded one if a respondent thinks the government is handling economy well and zero otherwise |
| *Health Handling* | How well or badly do you think the government is handling the following issues? “National Health Service”. A dummy variable coded one if a respondent thinks the government is handling NHS well and zero otherwise |
| ***Party voted for at last election*** |  |
| *Conservative* | Voted for Conservative Party in General Election in 2019 |
| *Labour* | Voted for Labour Party in General Election in 2019 |
| *Liberal* | Voted for Liberal democrats Party in General Election in 2019 |
| *SNP* | Voted for SNP in General Election in 2019 |
| *Other* | Voted for other party in General Election in 2019 |
| ***Covid-19 death*** | Number of reported Covid-19 related death |
| ***Demographic*** |  |
| *Age* | Age group of a respondent with the following categories: between 18 and 24 years old, between 25 and 49 years, 50-64 years old and over 65 years old |
| *Single* | A dummy variable taking value of one if a respondent is single and zero if in a relationship |
| *Female* | A dummy variable taking value of one if a respondent is single and zero if in a relationship |
| ***Socio-economic*** |  |
| *Educational level* | A categorical variable indicating whether an individual has a high, medium and low level of education achieved. |
| *Full time job* | A binary variable taking a value of one if an individual is in a full time employment |
| *Part time job* | A binary variable taking a value of one if an individual is in a part time employment |
| *Student* | A binary variable taking a value of one if an individual is a student |
| *Retired* | A binary variable taking a value of one if an individual is retired |
| *Unemployed* | A binary variable taking a value of one if an individual is unemployed |
| *Household income* | A categorical variable indicating whether an individual’s household earns below 35 thousand pounds, between 35 and 70 thousand pounds and over 70 thousand pounds |

# Appendix B: Summary statistics

### Table B1: Summary statistics before and after lockdown

|  | ***Before Lockdown*** | | | | | ***After Lockdown*** | | | | |  |
| --- | --- | --- | --- | --- | --- | --- | --- | --- | --- | --- | --- |
|  | (1) | (2) | (3) | (4) | (5) | (6) | (7) | (8) | (9) | (10) | (11) |
|  | N | mean | sd | min | max | N | mean | sd | min | max | p-value |
| Economy Handling | 15,649 | 0.519 | 0.500 | 0 | 1 | 18,105 | 0.569 | 0.495 | 0 | 1 | *** |
| Health Handling | 16,908 | 0.304 | 0.460 | 0 | 1 | 19,726 | 0.485 | 0.500 | 0 | 1 | *** |
| Covid death | 18,123 | 10.37 | 35.58 | 0 | 147 | 20,531 | 2,753 | 2,623 | 6 | 9,320 | *** |
| ***Demographic*** |  |  |  |  |  |  |  |  |  |  |  |
| Age | 18,123 | 2.754 | 0.984 | 1 | 4 | 20,531 | 2.756 | 0.955 | 1 | 4 |  |
| Single | 18,096 | 0.388 | 0.487 | 0 | 1 | 20,498 | 0.369 | 0.482 | 0 | 1 | *** |
| Female | 18,123 | 0.545 | 0.498 | 0 | 1 | 20,531 | 0.558 | 0.497 | 0 | 1 | ** |
| ***Socio-economic*** |  |  |  |  |  |  |  |  |  |  |  |
| Educational level | 18,123 | 1.965 | 0.755 | 1 | 3 | 20,531 | 2.031 | 0.748 | 1 | 3 | *** |
| Full time job | 17,697 | 0.377 | 0.485 | 0 | 1 | 19,844 | 0.393 | 0.488 | 0 | 1 | ** |
| Part time job | 17,697 | 0.141 | 0.348 | 0 | 1 | 19,844 | 0.141 | 0.348 | 0 | 1 |  |
| Student | 17,697 | 0.0387 | 0.193 | 0 | 1 | 19,844 | 0.0342 | 0.182 | 0 | 1 | * |
| Retired | 17,697 | 0.340 | 0.474 | 0 | 1 | 19,844 | 0.317 | 0.465 | 0 | 1 | *** |
| Unemployed | 17,697 | 0.104 | 0.305 | 0 | 1 | 19,844 | 0.115 | 0.319 | 0 | 1 | *** |
| Household income | 13,312 | 1.530 | 0.681 | 1 | 3 | 15,556 | 1.583 | 0.704 | 1 | 3 | *** |

*Note: One potential reason for the discrepancy between Figure 1 and the descriptive picture in Table B1 in appendix could arise because the descriptive table does not control for any confounding effect of other covariates at the individual and national level, nor of trends. This possibility is empirically confirmed by inspection of table C1 in the appendix that shows that lockdown has positive effect when controlling only for vote, but the sign of this effect then switches in subsequent columns when including trend, deaths and individual controls. Additional inspection, which we have carried out to address an anonymous reviewer’s comment, reveals that the coefficient switches from positive to negative as a result of controlling for the trend. It is also worth noting that Conservative voters represent 44.5% of our sample whereas Labour capture 33.2%; LibDem 12%; and SNP 4.2% (other 6.2%).*

### Table B2: Summary statistics before and after first UK Covid-19 death

|  | ***Excluding First Death*** | | | | | | ***First Death*** | | | | |  |
| --- | --- | --- | --- | --- | --- | --- | --- | --- | --- | --- | --- | --- |
| Column | 1 | 2 | 3 | 4 | 5 | 6 | | 7 | 8 | 9 | 10 | 11 |
|  | N | mean | sd | min | max | N | | mean | sd | min | max | p-value |
| Economy Handling | 32,591 | 0.546 | 0.498 | 0 | 1 | 1,163 | | 0.539 | 0.499 | 0 | 1 |  |
| Health Handling | 35,357 | 0.404 | 0.491 | 0 | 1 | 1,277 | | 0.331 | 0.471 | 0 | 1 | *** |
| Covid death | 37,306 | 1,520 | 2,376 | 0 | 9,320 | 1,348 | | 0.864 | 0.343 | 0 | 1 | *** |
| ***Demographic*** |  |  |  |  |  |  | |  |  |  |  |  |
| Age | 37,306 | 2.755 | 0.969 | 1 | 4 | 1,348 | | 2.747 | 0.965 | 1 | 4 |  |
| Single | 37,249 | 0.377 | 0.485 | 0 | 1 | 1,345 | | 0.384 | 0.486 | 0 | 1 |  |
| Female | 37,306 | 0.552 | 0.497 | 0 | 1 | 1,348 | | 0.556 | 0.497 | 0 | 1 |  |
| ***Socio-economic*** |  |  |  |  |  |  | |  |  |  |  |  |
| Educational level | 37,306 | 2.000 | 0.752 | 1 | 3 | 1,348 | | 1.988 | 0.746 | 1 | 3 | * |
| Full time job | 36,226 | 0.386 | 0.487 | 0 | 1 | 1,315 | | 0.382 | 0.486 | 0 | 1 |  |
| Part time job | 36,226 | 0.141 | 0.348 | 0 | 1 | 1,315 | | 0.144 | 0.351 | 0 | 1 |  |
| Student | 36,226 | 0.0359 | 0.186 | 0 | 1 | 1,315 | | 0.0479 | 0.214 | 0 | 1 | * |
| Retired | 36,226 | 0.328 | 0.469 | 0 | 1 | 1,315 | | 0.326 | 0.469 | 0 | 1 |  |
| Unemployed | 36,226 | 0.110 | 0.313 | 0 | 1 | 1,315 | | 0.100 | 0.301 | 0 | 1 |  |
| Household income | 27,920 | 1.558 | 0.694 | 1 | 3 | 948 | | 1.575 | 0.691 | 1 | 3 |  |

### Table B3: Summary statistics before and after Boris Johnson’s hospitalization

|  | ***Excluding Boris Johnson Hospitalization*** | | | | | ***Boris Johnson in Hospital*** | | | | |  |
| --- | --- | --- | --- | --- | --- | --- | --- | --- | --- | --- | --- |
| Column | 1 | 2 | 3 | 4 | 5 | 6 | 7 | 8 | 9 | 10 | 11 |
|  | N | mean | sd | min | max | N | mean | sd | min | max | p-value |
| Economy Handling | 32,605 | 0.543 | 0.498 | 0 | 1 | 1,149 | 0.62 | 0.486 | 0 | 1 | *** |
| Health Handling | 35,339 | 0.397 | 0.489 | 0 | 1 | 1,295 | 0.519 | 0.5 | 0 | 1 | *** |
| Covid death | 37,314 | 1,393 | 2,345 | 0 | 9,320 | 1,340 | 3,531 | 1,387 | 162 | 4,103 | *** |
| ***Demographic*** |  |  |  |  |  |  |  |  |  |  |  |
| Age | 37,314 | 2.753 | 0.969 | 1 | 4 | 1,340 | 2.81 | 0.951 | 1 | 4 |  |
| Single | 37,259 | 0.378 | 0.485 | 0 | 1 | 1,335 | 0.364 | 0.481 | 0 | 1 |  |
| Female | 37,314 | 0.552 | 0.497 | 0 | 1 | 1,340 | 0.55 | 0.498 | 0 | 1 |  |
| ***Socio-economic*** |  |  |  |  |  |  |  |  |  |  |  |
| Educational level | 37,314 | 1.999 | 0.753 | 1 | 3 | 1,340 | 2.033 | 0.741 | 1 | 3 |  |
| Full time job | 36,241 | 0.386 | 0.487 | 0 | 1 | 1,300 | 0.378 | 0.485 | 0 | 1 |  |
| Part time job | 36,241 | 0.140 | 0.347 | 0 | 1 | 1,300 | 0.158 | 0.365 | 0 | 1 |  |
| Student | 36,241 | 0.0365 | 0.188 | 0 | 1 | 1,300 | 0.0315 | 0.175 | 0 | 1 |  |
| Retired | 36,241 | 0.327 | 0.469 | 0 | 1 | 1,300 | 0.345 | 0.476 | 0 | 1 |  |
| Unemployed | 36,241 | 0.110 | 0.313 | 0 | 1 | 1,300 | 0.0862 | 0.281 | 0 | 1 | ** |
| Household income | 27,860 | 1.558 | 0.694 | 1 | 3 | 1,008 | 1.564 | 0.689 | 1 | 3 |  |

### Table B4: Summary statistics before and after Dominic Cumming's non-compliance with lockdown regulations

|  | ***Excluding Dominic Cumming's Event*** | | | | | ***Dominic Cumming's Event*** | | | | |  |
| --- | --- | --- | --- | --- | --- | --- | --- | --- | --- | --- | --- |
| Column | 1 | 2 | 3 | 4 | 5 | 6 | 7 | 8 | 9 | 10 | 11 |
|  | N | mean | sd | min | max | N | mean | sd | min | max | p-value |
| Economy Handling | 32,508 | 0.546 | 0.498 | 0 | 1 | 1,246 | 0.533 | 0.499 | 0 | 1 |  |
| Health Handling | 35,279 | 0.398 | 0.490 | 0 | 1 | 1,355 | 0.476 | 0.500 | 0 | 1 | *** |
| Covid death | 37,246 | 1,458 | 2,391 | 0 | 9,320 | 1,408 | 1,711 | 633.2 | 98 | 1,966 | *** |
| ***Demographic*** |  |  |  |  |  |  |  |  |  |  |  |
| Age | 37,246 | 2.755 | 0.969 | 1 | 4 | 1,408 | 2.763 | 0.950 | 1 | 4 |  |
| Single | 37,188 | 0.378 | 0.485 | 0 | 1 | 1,406 | 0.370 | 0.483 | 0 | 1 |  |
| Female | 37,246 | 0.552 | 0.497 | 0 | 1 | 1,408 | 0.556 | 0.497 | 0 | 1 |  |
| ***Socio-economic*** |  |  |  |  |  |  |  |  |  |  |  |
| Educational level | 37,246 | 1.998 | 0.752 | 1 | 3 | 1,408 | 2.042 | 0.761 | 1 | 3 |  |
| Full time job | 36,171 | 0.385 | 0.487 | 0 | 1 | 1,370 | 0.399 | 0.490 | 0 | 1 |  |
| Part time job | 36,171 | 0.141 | 0.348 | 0 | 1 | 1,370 | 0.136 | 0.343 | 0 | 1 |  |
| Student | 36,171 | 0.0365 | 0.187 | 0 | 1 | 1,370 | 0.0328 | 0.178 | 0 | 1 |  |
| Retired | 36,171 | 0.328 | 0.470 | 0 | 1 | 1,370 | 0.312 | 0.463 | 0 | 1 |  |
| Unemployed | 36,171 | 0.109 | 0.312 | 0 | 1 | 1,370 | 0.120 | 0.325 | 0 | 1 |  |
| Household income | 27,815 | 1.557 | 0.694 | 1 | 3 | 1,053 | 1.600 | 0.697 | 1 | 3 | * |

### Table B5: Mean and standard deviation of answers to questions about handling of economy and health by labour versus conservative voters

| Issue (right) | **Economy** | | **Health** | |
| --- | --- | --- | --- | --- |
| Party (below) | Mean | SD | Mean | SD |
| Conservative | 0.84 | 0.37 | 0.69 | 0.46 |
| Labour | 0.24 | 0.43 | 0.12 | 0.33 |

### Table B6: Monthly average of answers to questions about handling of economy and health by labour versus conservative voters

| **Issue (right)** | **Economy** | | **Health** | |
| --- | --- | --- | --- | --- |
| **Party (right) and Date (below)** | **Conservative** | **Labour** | **Conservative** | **Labour** |
| June 2019 | 0.67 | 0.12 | 0.38 | 0.04 |
| July 2019 | 0.7 | 0.15 | 0.37 | 0.07 |
| August 2019 | 0.7 | 0.17 | 0.44 | 0.1 |
| September 2019 | 0.76 | 0.15 | 0.52 | 0.09 |
| October 2019 | 0.72 | 0.14 | 0.49 | 0.08 |
| November 2019 | 0.79 | 0.17 | 0.51 | 0.06 |
| December 2019 | 0.82 | 0.18 | 0.51 | 0.04 |
| January 2020 | 0.87 | 0.18 | 0.52 | 0.07 |
| February 2020 | 0.88 | 0.19 | 0.53 | 0.05 |
| **March 2020 (lockdown)** | **0.89** | **0.25** | **0.71** | **0.12** |
| April 2020 | 0.88 | 0.3 | 0.81 | 0.18 |
| May 2020 | 0.85 | 0.29 | 0.81 | 0.17 |
| June 2020 | 0.81 | 0.25 | 0.78 | 0.15 |

### Table B7. Summary statistics of individuals with different last vote, before and after each event

|  | | ***Before Lockdown*** | | | | | | | | ***After Lockdown*** | | | | |  |
| --- | --- | --- | --- | --- | --- | --- | --- | --- | --- | --- | --- | --- | --- | --- | --- |
|  | | (1) | | (2) | | (3) | | (4) | (5) | (6) | (7) | (8) | (9) | (10) | (11) |
|  | | N | | mean | | sd | | min | max | N | mean | sd | min | max | p-value |
| *Conservative* | | 18,123 | | 0.447 | | 0.497 | | 0 | 1 | 20,531 | 0.444 | 0.497 | 0 | 1 |  |
| *Labour* | | 18,123 | | 0.336 | | 0.472 | | 0 | 1 | 20,531 | 0.329 | 0.470 | 0 | 1 |  |
| *Liberal* | | 18,123 | | 0.117 | | 0.321 | | 0 | 1 | 20,531 | 0.123 | 0.329 | 0 | 1 |  |
| *SNP* | | 18,123 | | 0.0415 | | 0.200 | | 0 | 1 | 20,531 | 0.0416 | 0.200 | 0 | 1 |  |
| *Other* | | 18,123 | | 0.0591 | | 0.236 | | 0 | 1 | 20,531 | 0.0616 | 0.240 | 0 | 1 |  |
|  | | ***Excluding First Death*** | | | | | | | | ***After First Death*** | | | | |  |
| *Conservative* | | 37,306 | | 0.446 | | 0.497 | | 0 | 1 | 1,348 | 0.438 | 0.496 | 0 | 1 |  |
| *Labour* | | 37,306 | | 0.332 | | 0.471 | | 0 | 1 | 1,348 | 0.335 | 0.472 | 0 | 1 |  |
| *Liberal* | | 37,306 | | 0.120 | | 0.324 | | 0 | 1 | 1,348 | 0.142 | 0.350 | 0 | 1 | * |
| *SNP* | | 37,306 | | 0.0418 | | 0.200 | | 0 | 1 | 1,348 | 0.0356 | 0.185 | 0 | 1 |  |
| *Other* | | 37,306 | | 0.0609 | | 0.239 | | 0 | 1 | 1,348 | 0.0482 | 0.214 | 0 | 1 |  |
|  | ***Excluding Boris Johnson Hospitalization*** | | | | | | | | | ***After Boris Johnson in Hospital*** | | | | |  |
| *Conservative* | 37,314 | | 0.446 | | 0.497 | | 0 | | 1 | 1,340 | 0.438 | 0.496 | 0 | 1 |  |
| *Labour* | 37,314 | | 0.332 | | 0.471 | | 0 | | 1 | 1,340 | 0.344 | 0.475 | 0 | 1 |  |
| *Liberal* | 37,314 | | 0.120 | | 0.325 | | 0 | | 1 | 1,340 | 0.117 | 0.322 | 0 | 1 |  |
| *SNP* | 37,314 | | 0.0416 | | 0.200 | | 0 | | 1 | 1,340 | 0.0418 | 0.2 | 0 | 1 |  |
| *Other* | 37,314 | | 0.0605 | | 0.238 | | 0 | | 1 | 1,340 | 0.059 | 0.236 | 0 | 1 |  |
|  | ***Before Dominic Cumming's Event*** | | | | | | | | | ***After Dominic Cumming's Event*** | | | | |  |
| *Conservative* | 37,246 | | 0.446 | | 0.497 | | 0 | | 1 | 1,408 | 0.440 | 0.497 | 0 | 1 |  |
| *Labour* | 37,246 | | 0.332 | | 0.471 | | 0 | | 1 | 1,408 | 0.341 | 0.474 | 0 | 1 |  |
| *Liberal* | 37,246 | | 0.120 | | 0.325 | | 0 | | 1 | 1,408 | 0.118 | 0.323 | 0 | 1 |  |
| *SNP* | 37,246 | | 0.0417 | | 0.200 | | 0 | | 1 | 1,408 | 0.0369 | 0.189 | 0 | 1 |  |
| *Other* | 37,246 | | 0.0603 | | 0.238 | | 0 | | 1 | 1,408 | 0.0639 | 0.245 | 0 | 1 |  |

### Figure B1: Google trends for search terms Cummings and Johnson in 2020

*Source: Google trends UK.*

### Figure B2. Most important issues for conservative excluding labour voters

# Appendix C: Full results

### Table C1: Full results for government handling of economy

| Column | 1 | 2 | 3 | 4 | 5 | 6 | 7 | 8 | 9 |
| --- | --- | --- | --- | --- | --- | --- | --- | --- | --- |
| MODEL | Demographic | Demographic | Demographic | Socio-economic | Socio-economic | Socio-economic | Covid | Covid | Full |
| Labour | -0.600*** | -0.598*** | -0.578*** | -0.587*** | -0.585*** | -0.574*** | -0.598*** | -0.599*** | -0.564*** |
| Liberal Dem | -0.450*** | -0.449*** | -0.438*** | -0.445*** | -0.448*** | -0.436*** | -0.447*** | -0.449*** | -0.431*** |
| SNP | -0.643*** | -0.641*** | -0.626*** | -0.632*** | -0.639*** | -0.632*** | -0.622*** | -0.622*** | -0.604*** |
| Other | -0.341*** | -0.339*** | -0.330*** | -0.334*** | -0.326*** | -0.319*** | -0.336*** | -0.338*** | -0.317*** |
| Female |  | -0.0110** | -0.0108** |  |  |  |  |  | -0.00916 |
| Single |  | -0.0130** | -0.00975* |  |  |  |  |  | 0.00741 |
| Age = 2, 25-49 |  |  | -0.0220** |  |  |  |  |  | 0.000908 |
| Age = 3, 50-64 |  |  | 0.0335*** |  |  |  |  |  | 0.0582*** |
| Age = 4, 65+ |  |  | 0.0446*** |  |  |  |  |  | 0.0646*** |
| Work status: Part time |  |  |  | 0.0102 | 0.0216*** | 0.0191** |  |  | 0.0119 |
| Work status: Student |  |  |  | 0.0133 | 0.0283 | 0.0264 |  |  | 0.0406* |
| Work status: Retired |  |  |  | 0.0379*** | 0.0428*** | 0.0372*** |  |  | -0.000730 |
| Work status: Unemployed |  |  |  | -0.0526*** | -0.0432*** | -0.0495*** |  |  | -0.0578*** |
| Educational level: Medium |  |  |  |  |  | -0.0164*** |  |  | -0.0172*** |
| Educational level: High |  |  |  |  |  | -0.0605*** |  |  | -0.0584*** |
| Household income: 35K-70K |  |  |  |  | 0.0174** | 0.0244*** |  |  | 0.0253*** |
| Household income: >70K |  |  |  |  | 0.0354*** | 0.0470*** |  |  | 0.0515*** |
| Rest of South |  |  |  |  |  |  | 0.0346*** | 0.0336*** | 0.0201 |
| Midlands |  |  |  |  |  |  | 0.0524*** | 0.0523*** | 0.0452*** |
| North |  |  |  |  |  |  | 0.0434*** | 0.0422*** | 0.0400*** |
| Scotland |  |  |  |  |  |  | 0.0137 | 0.0251 | 0.0202 |
| Wales |  |  |  |  |  |  | 0.000630 | 0.0134 | 0.0209 |
| Covid-19 Death |  |  |  |  |  |  |  | 8.05e-06*** | 8.54e-06*** |
| Trend |  |  |  |  |  |  |  | 0.00220*** | 0.00237*** |
| Observations | 33,754 | 33,704 | 33,704 | 32,811 | 25,057 | 25,057 | 33,754 | 33,754 | 25,045 |
| R-squared | 0.314 | 0.314 | 0.318 | 0.317 | 0.314 | 0.316 | 0.315 | 0.321 | 0.325 |

*Note: Robust standard errors; *** p<0.01, ** p<0.05, * p<0.1 All models include a constant.*

### Table C1 (cont.): Full results for government handling of economy

| Column | 10 | 11 | 12 | 13 | 14 | 15 | 16 | 17 |
| --- | --- | --- | --- | --- | --- | --- | --- | --- |
| MODEL | First death | Lockdown | Boris Hospital | Cummings | First death + C | Lockdown + C | Boris Hospital + C | Cummings + C |
| Labour | -0.600*** | -0.600*** | -0.600*** | -0.600*** | -0.564*** | -0.564*** | -0.564*** | -0.564*** |
| Liberal Dem | -0.450*** | -0.451*** | -0.450*** | -0.450*** | -0.431*** | -0.432*** | -0.431*** | -0.431*** |
| SNP | -0.643*** | -0.643*** | -0.643*** | -0.643*** | -0.604*** | -0.605*** | -0.604*** | -0.604*** |
| Other | -0.341*** | -0.342*** | -0.341*** | -0.341*** | -0.317*** | -0.318*** | -0.318*** | -0.318*** |
| Female |  |  |  |  | 0.00736 | -0.00926 | -0.00916 | -0.00916 |
| Single |  |  |  |  | -0.00917 | 0.00736 | 0.00747 | 0.00744 |
| Age = 2, 25-49 |  |  |  |  | 0.000868 | 0.000211 | 0.000397 | 0.000809 |
| Age = 3, 50-64 |  |  |  |  | 0.0581*** | 0.0573*** | 0.0578*** | 0.0582*** |
| Age = 4, 65+ |  |  |  |  | 0.0646*** | 0.0641*** | 0.0641*** | 0.0645*** |
| Work status: Part time |  |  |  |  | 0.0118 | 0.0121 | 0.0117 | 0.0118 |
| Work status: Student |  |  |  |  | 0.0404* | 0.0390* | 0.0404* | 0.0406* |
| Work status: Retired |  |  |  |  | -0.000750 | -0.000973 | -0.00103 | -0.000779 |
| Work status: Unemployed |  |  |  |  | -0.0578*** | -0.0574*** | -0.0578*** | -0.0579*** |
| Educational level: Medium |  |  |  |  | -0.0172*** | -0.0173*** | -0.0173*** | -0.0173*** |
| Educational level: High |  |  |  |  | -0.0584*** | -0.0588*** | -0.0585*** | -0.0585*** |
| Household income: 35K-70K |  |  |  |  | 0.0252*** | 0.0250*** | 0.0251*** | 0.0253*** |
| Household income: >70K |  |  |  |  | 0.0515*** | 0.0511*** | 0.0515*** | 0.0514*** |
| Rest of South |  |  |  |  | 0.0201 | 0.0199 | 0.0201 | 0.0200 |
| Midlands |  |  |  |  | 0.0452*** | 0.0451*** | 0.0450*** | 0.0450*** |
| North |  |  |  |  | 0.0399*** | 0.0396*** | 0.0400*** | 0.0399*** |
| Scotland |  |  |  |  | 0.0204 | 0.0249 | 0.0191 | 0.0199 |
| Wales |  |  |  |  | 0.0211 | 0.0258* | 0.0199 | 0.0207 |
| Covid-19 Death |  |  |  |  | 8.70e-06*** | 1.16e-05*** | 7.89e-06*** | 8.46e-06*** |
| Trend |  |  |  |  | 0.00236*** | 0.00332*** | 0.00238*** | 0.00244*** |
| First UK Death | -0.00498 |  |  |  | 0.0154 |  |  |  |
| Lockdown |  | 0.0521*** |  |  |  | -0.0368** |  |  |
| BJ Hospitalised |  |  | 0.0737*** |  |  |  | 0.0450** |  |
| Cummings |  |  |  | -0.00599 |  |  |  | -0.0286** |
| Observations | 33,754 | 33,754 | 33,754 | 33,754 | 25,045 | 25,045 | 25,045 | 25,045 |
| R-squared | 0.314 | 0.317 | 0.315 | 0.314 | 0.326 | 0.326 | 0.326 | 0.326 |

*Note: Robust standard errors; *** p<0.01, ** p<0.05, * p<0.1 All models include a constant.*

### Table C1 (cont.): Full results for government handling of economy

| Column | 18 | 19 | 20 | 21 |
| --- | --- | --- | --- | --- |
| MODEL | First death + CI | Lockdown + CI | Boris Hospital + CI | Cummings + CI |
| Labour | -0.561*** | -0.603*** | -0.564*** | -0.565*** |
| Liberal Dem | -0.602*** | -0.499*** | -0.434*** | -0.433*** |
| SNP | -0.602*** | -0.654*** | -0.602*** | -0.609*** |
| Other | -0.321*** | -0.326*** | -0.320*** | -0.320*** |
| Female | -0.00905 | -0.00945* | -0.00910 | -0.00914 |
| Single | 0.00727 | 0.00770 | 0.00747 | 0.00755 |
| Age = 2, 25-49 | 0.00138 | -0.000495 | 0.000423 | 0.000675 |
| Age = 3, 50-64 | 0.0583*** | 0.0559*** | 0.0577*** | 0.0581*** |
| Age = 4, 65+ | 0.0644*** | 0.0622*** | 0.0639*** | 0.0642*** |
| Work status: Part time | 0.0120 | 0.0129 | 0.0117 | 0.0121 |
| Work status: Student | 0.0417** | 0.0409* | 0.0404* | 0.0404* |
| Work status: Retired | -0.000324 | -0.00155 | -0.000858 | -0.000517 |
| Work status: Unemployed | -0.0570*** | -0.0577*** | -0.0579*** | -0.0579*** |
| Educational level: Medium | -0.0174*** | -0.0179*** | -0.0173*** | -0.0173*** |
| Educational level: High | -0.0584*** | -0.0594*** | -0.0586*** | -0.0586*** |
| Household income: 35K-70K | 0.0251*** | 0.0250*** | 0.0251*** | 0.0255*** |
| Household income: >70K | 0.0512*** | 0.0505*** | 0.0516*** | 0.0517*** |
| Rest of South | 0.0203 | 0.0186 | 0.0203 | 0.0198 |
| Midlands | 0.0455*** | 0.0440*** | 0.0451*** | 0.0448*** |
| North | 0.0399*** | 0.0383*** | 0.0399*** | 0.0398*** |
| Scotland | 0.0180 | 0.0245 | 0.0191 | 0.0198 |
| Wales | 0.0196 | 0.0258* | 0.0198 | 0.0207 |
| Covid-19 Death | 7.44e-06*** | 1.20e-05*** | 7.84e-06*** | 8.47e-06*** |
| Trend | 0.00240*** | 0.00340*** | 0.00239*** | 0.00244*** |
| First UK Death | 0.0576** |  |  |  |
| Labour*First Death | -0.0878*** |  |  |  |
| Liberal Dem*First Death | -0.118*** |  |  |  |
| SNP*First Death | -0.0944*** |  |  |  |
| Other*First Death | 0.127*** |  |  |  |
| Lockdown |  | -0.0826*** |  |  |
| Labour*Lockdown |  | 0.0718*** |  |  |
| Liberal Dem*Lockdown |  | 0.122*** |  |  |
| SNP*Lockdown |  | 0.0905*** |  |  |
| Other*Lockdown |  | 0.0171 |  |  |
| BJ Hospitalised |  |  | 0.0293*** |  |
| Labour*BJ |  |  | 0.0145 |  |
| Liberal Dem*BJ |  |  | 0.0793*** |  |
| SNP*BJ |  |  | -0.0814*** |  |
| Other*BJ |  |  | 0.0748 |  |
| Cummings |  |  |  | -0.0503*** |
| Labour*Cummings |  |  |  | 0.0198 |
| Liberal Dem*Cummings |  |  |  | 0.0525 |
| SNP*Cummings |  |  |  | 0.133*** |
| Other*Cummings |  |  |  | 0.0572 |
| Observations | 25,045 | 25,045 | 25,045 | 25,045 |
| R-squared | 0.326 | 0.328 | 0.326 | 0.326 |

*Note: Robust standard errors; *** p<0.01, ** p<0.05, * p<0.1 All models include a constant.*

### Table C2: Full results for government handling of health

| Column | 1 | 2 | 3 | 4 | 5 | 6 | 7 | 8 | 9 |
| --- | --- | --- | --- | --- | --- | --- | --- | --- | --- |
| MODEL | Demographic | Demographic | Demographic | Socio-economic | Socio-economic | Socio-economic | Covid | Covid | Full |
| Labour | -0.562*** | -0.558*** | -0.539*** | -0.547*** | -0.552*** | -0.541*** | -0.560*** | -0.562*** | -0.533*** |
| Liberal Dem | -0.446*** | -0.445*** | -0.434*** | -0.439*** | -0.444*** | -0.433*** | -0.444*** | -0.451*** | -0.431*** |
| SNP | -0.543*** | -0.540*** | -0.525*** | -0.531*** | -0.535*** | -0.528*** | -0.550*** | -0.550*** | -0.532*** |
| Other | -0.350*** | -0.347*** | -0.338*** | -0.342*** | -0.344*** | -0.338*** | -0.347*** | -0.349*** | -0.338*** |
| Female |  | -0.0154*** | -0.0147*** |  |  |  |  |  | -0.0275*** |
| Single |  | -0.0214*** | -0.0167*** |  |  |  |  |  | -0.00641 |
| Age = 2, 25-49 |  |  | -0.00269 |  |  |  |  |  | -0.0181 |
| Age = 3, 50-64 |  |  | 0.0420*** |  |  |  |  |  | 0.0102 |
| Age = 4, 65+ |  |  | 0.0552*** |  |  |  |  |  | 0.0203 |
| Work status: Part time |  |  |  | 0.0192*** | 0.0132* | 0.0112 |  |  | 0.0140* |
| Work status: Student |  |  |  | -0.0196* | -0.0179 | -0.0215 |  |  | -0.0146 |
| Work status: Retired |  |  |  | 0.0484*** | 0.0417*** | 0.0369*** |  |  | 0.0140 |
| Work status: Unemployed |  |  |  | 0.0112 | 0.0192** | 0.0135 |  |  | 0.00277 |
| Educational level: Medium |  |  |  |  |  | -0.00807 |  |  | -0.0170** |
| Educational level: High |  |  |  |  |  | -0.0519*** |  |  | -0.0609*** |
| Household income: 35K-70K |  |  |  |  | 0.0132** | 0.0190*** |  |  | 0.0103* |
| Household income: >70K |  |  |  |  | 0.00583 | 0.0155* |  |  | 0.00395 |
| Rest of South |  |  |  |  |  |  | 0.0235 | 0.0195 | 0.000246 |
| Midlands |  |  |  |  |  |  | 0.0339 | 0.0319** | 0.0182 |
| North |  |  |  |  |  |  | 0.0312 | 0.0273* | 0.0160 |
| Scotland |  |  |  |  |  |  | 0.0311 | 0.0484*** | 0.0416** |
| Wales |  |  |  |  |  |  | -0.00137 | 0.0192 | 0.0161 |
| Covid-19 Death |  |  |  |  |  |  |  | 1.41e-05*** | 1.50e-05*** |
| Trend |  |  |  |  |  |  |  | 0.00590*** | 0.00613*** |
| Observations | 36,634 | 36,578 | 36,578 | 35,600 | 26,959 | 26,959 | 36,634 | 36,634 | 26,945 |
| R-squared | 0.282 | 0.283 | 0.285 | 0.284 | 0.284 | 0.286 | 0.283 | 0.316 | 0.322 |

*Note: Robust standard errors; *** p<0.01, ** p<0.05, * p<0.1 All models include a constant.*

### Table C2 (cont.): Full results for government handling of health

| Column | 10 | 11 | 12 | 13 | 14 | 15 | 16 | 17 |
| --- | --- | --- | --- | --- | --- | --- | --- | --- |
| MODEL | First death | Lockdown | Boris Hospital | Cummings | First death + C | Lockdown + C | Boris Hospital + C | Cummings + C |
| Labour | -0.562*** | -0.561*** | -0.562*** | -0.562*** | -0.533*** | -0.532*** | -0.534*** | -0.533*** |
| Liberal Dem | -0.445*** | -0.449*** | -0.446*** | -0.446*** | -0.430*** | -0.429*** | -0.431*** | -0.431*** |
| SNP | -0.543*** | -0.543*** | -0.543*** | -0.543*** | -0.532*** | -0.530*** | -0.532*** | -0.532*** |
| Other | -0.351*** | -0.352*** | -0.350*** | -0.350*** | -0.339*** | -0.338*** | -0.339*** | -0.338*** |
| Female |  |  |  |  | -0.0274*** | -0.0270*** | -0.0275*** | -0.0275*** |
| Single |  |  |  |  | -0.00630 | -0.00633 | -0.00638 | -0.00642 |
| Age = 2, 25-49 |  |  |  |  | -0.0179 | -0.0163 | -0.0189 | -0.0181 |
| Age = 3, 50-64 |  |  |  |  | 0.0104 | 0.0124 | 0.00935 | 0.0101 |
| Age = 4, 65+ |  |  |  |  | 0.0203 | 0.0214 | 0.0195 | 0.0203 |
| Work status: Part time |  |  |  |  | 0.0142* | 0.0135* | 0.0139* | 0.0140* |
| Work status: Student |  |  |  |  | -0.0143 | -0.0110 | -0.0148 | -0.0146 |
| Work status: Retired |  |  |  |  | 0.0141 | 0.0148 | 0.0135 | 0.0140 |
| Work status: Unemployed |  |  |  |  | 0.00266 | 0.00159 | 0.00290 | 0.00278 |
| Educational level: Medium |  |  |  |  | -0.0170** | -0.0165** | -0.0171** | -0.0170** |
| Educational level: High |  |  |  |  | -0.0611*** | -0.0599*** | -0.0612*** | -0.0609*** |
| Household income: 35K-70K |  |  |  |  | 0.0105* | 0.0109* | 0.0101* | 0.0102* |
| Household income: >70K |  |  |  |  | 0.00412 | 0.00494 | 0.00397 | 0.00396 |
| Rest of South |  |  |  |  | 0.000365 | 0.000867 | 0.000107 | 0.000289 |
| Midlands |  |  |  |  | 0.0183 | 0.0186 | 0.0178 | 0.0183 |
| North |  |  |  |  | 0.0162 | 0.0166 | 0.0160 | 0.0160 |
| Scotland |  |  |  |  | 0.0410** | 0.0282 | 0.0398** | 0.0417** |
| Wales |  |  |  |  | 0.0157 | 0.00203 | 0.0141 | 0.0162 |
| Covid-19 Death |  |  |  |  | 1.46e-05*** | 6.32e-06*** | 1.38e-05*** | 1.50e-05*** |
| Trend |  |  |  |  | 0.00614*** | 0.00344*** | 0.00616*** | 0.00610*** |
| First UK Death | -0.0644*** |  |  |  | -0.0410*** |  |  |  |
| Lockdown |  | 0.181*** |  |  |  | 0.104*** |  |  |
| BJ Hospitalised |  |  | 0.126*** |  |  |  | 0.0771*** |  |
| Cummings |  |  |  | 0.0801*** |  |  |  | 0.0106 |
| Observations | 36,634 | 36,634 | 36,634 | 36,634 | 26,945 | 26,945 | 26,945 | 26,945 |
| R-squared | 0.283 | 0.316 | 0.285 | 0.283 | 0.323 | 0.326 | 0.323 | 0.323 |

*Note: Robust standard errors; *** p<0.01, ** p<0.05, * p<0.1 All models include a constant.*

### Table C2 (cont.): Full results for government handling of health

| Column | 18 | 19 | 20 | 21 |
| --- | --- | --- | --- | --- |
| MODEL | First death + CI | Lockdown + CI | Boris Hospital + CI | Cummings + CI |
| Labour | -0.534*** | -0.445*** | -0.531*** | -0.530*** |
| Liberal Dem | -0.431*** | -0.387*** | -0.430*** | -0.425*** |
| SNP | -0.534*** | -0.447*** | -0.530*** | -0.531*** |
| Other | -0.342*** | -0.276*** | -0.337*** | -0.334*** |
| Female | -0.0274*** | -0.0271*** | -0.0275*** | -0.0275*** |
| Single | -0.00626 | -0.00639 | -0.00631 | -0.00651 |
| Age = 2, 25-49 | -0.0180 | -0.0139 | -0.0187 | -0.0177 |
| Age = 3, 50-64 | 0.0102 | 0.0154 | 0.00962 | 0.0103 |
| Age = 4, 65+ | 0.0201 | 0.0252 | 0.0198 | 0.0208 |
| Work status: Part time | 0.0141* | 0.0127* | 0.0139* | 0.0140* |
| Work status: Student | -0.0142 | -0.0117 | -0.0146 | -0.0139 |
| Work status: Retired | 0.0142 | 0.0158 | 0.0135 | 0.0140 |
| Work status: Unemployed | 0.00264 | 0.00230 | 0.00278 | 0.00327 |
| Educational level: Medium | -0.0171** | -0.0156** | -0.0171** | -0.0170** |
| Educational level: High | -0.0611*** | -0.0593*** | -0.0609*** | -0.0608*** |
| Household income: 35K-70K | 0.0104* | 0.0118** | 0.0101* | 0.0102* |
| Household income: >70K | 0.00408 | 0.00565 | 0.00407 | 0.00410 |
| Rest of South | 0.000357 | 0.00329 | 0.000337 | 0.000694 |
| Midlands | 0.0183 | 0.0205 | 0.0179 | 0.0187 |
| North | 0.0162 | 0.0188 | 0.0163 | 0.0163 |
| Scotland | 0.0411** | 0.0279 | 0.0399** | 0.0421** |
| Wales | 0.0157 | 0.00164 | 0.0144 | 0.0163 |
| Covid-19 Death | 1.45e-05*** | 5.40e-06** | 1.38e-05*** | 1.50e-05*** |
| Trend | 0.00615*** | 0.00328*** | 0.00616*** | 0.00610*** |
| First UK Death | -0.0539*** |  |  |  |
| Labour*First Death | 0.00948 |  |  |  |
| Liberal Dem*First Death | 0.0146 |  |  |  |
| SNP*First Death | 0.0562** |  |  |  |
| Other*First Death | 0.110 |  |  |  |
| Lockdown |  | 0.186*** |  |  |
| Labour*Lockdown |  | -0.160*** |  |  |
| Liberal Dem*Lockdown |  | -0.0766*** |  |  |
| SNP*Lockdown |  | -0.151*** |  |  |
| Other*Lockdown |  | -0.112*** |  |  |
| BJ Hospitalised |  |  | 0.105*** |  |
| Labour*BJ |  |  | -0.0632** |  |
| Liberal Dem*BJ |  |  | -0.00497 |  |
| SNP*BJ |  |  | -0.0548*** |  |
| Other*BJ |  |  | -0.0542 |  |
| Cummings |  |  |  | 0.0627*** |
| Labour*Cummings |  |  |  | -0.0770*** |
| Liberal Dem*Cummings |  |  |  | -0.147*** |
| SNP*Cummings |  |  |  | -0.0170 |
| Other*Cummings |  |  |  | -0.116** |
| Observations | 26,945 | 26,945 | 26,945 | 26,945 |
| R-squared | 0.323 | 0.331 | 0.323 | 0.323 |

*Note: Robust standard errors; *** p<0.01, ** p<0.05, * p<0.1 All models include a constant.*

# Appendix D. Robustness checks.

In order to investigate whether our results could be due to the reducing case numbers and the early stages of opening up captured by the June 2020 data, we have re-run our analysis for Cummings’ scandal excluding June’s information. The results are provided below and they look very similar to the results which include June’s data. We have also rerun our analysis for the Cummings’ scandal while controlling for hospitalization, measured as weekly numbers of COVID-19 patients admitted to hospital. We are grateful to an anonymous reviewer for suggesting these robustness checks. Moreover, we have also reproduced our analysis using logistic regression analysis instead of linear probability models: all our findings are similar, except for the effect of lockdown on perceptions of government handling of the economy, where the effect remains negative and significant for Conservative voters, but is no longer statistically significant for Labour and LibDem voters.

## Additional tests

### Figure D1: Impact of events on perceptions of government handling of health and economy

*
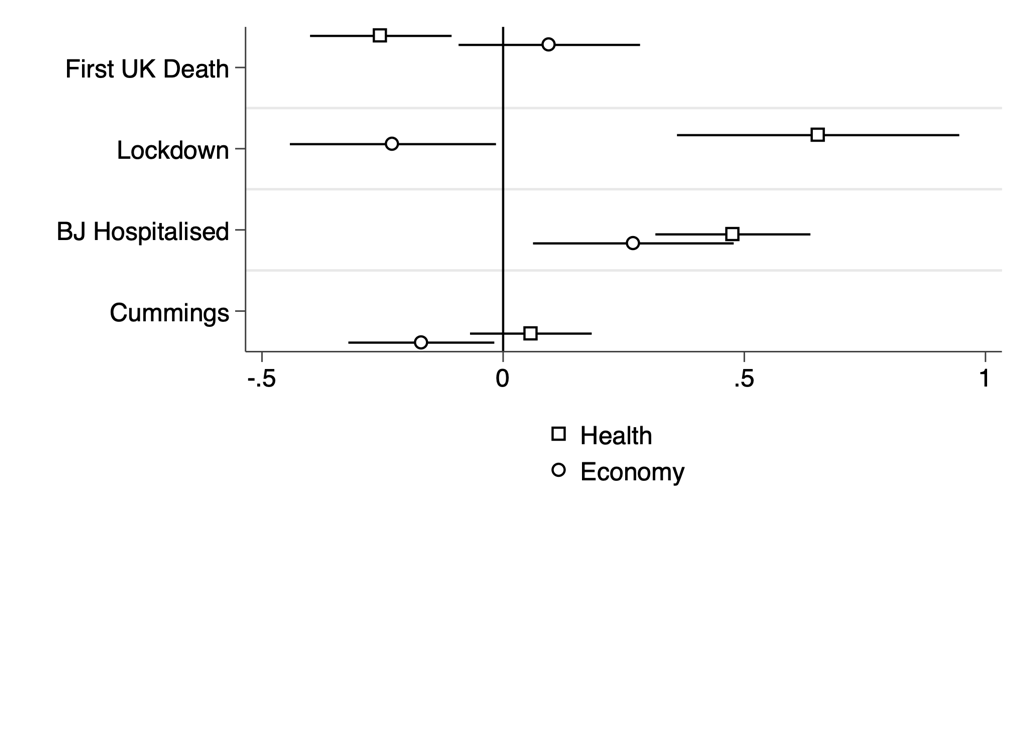
*

### Figure D2: Impact of events on perceptions of government handling of health and economy for Scotland and England.

Panel A: Scotland
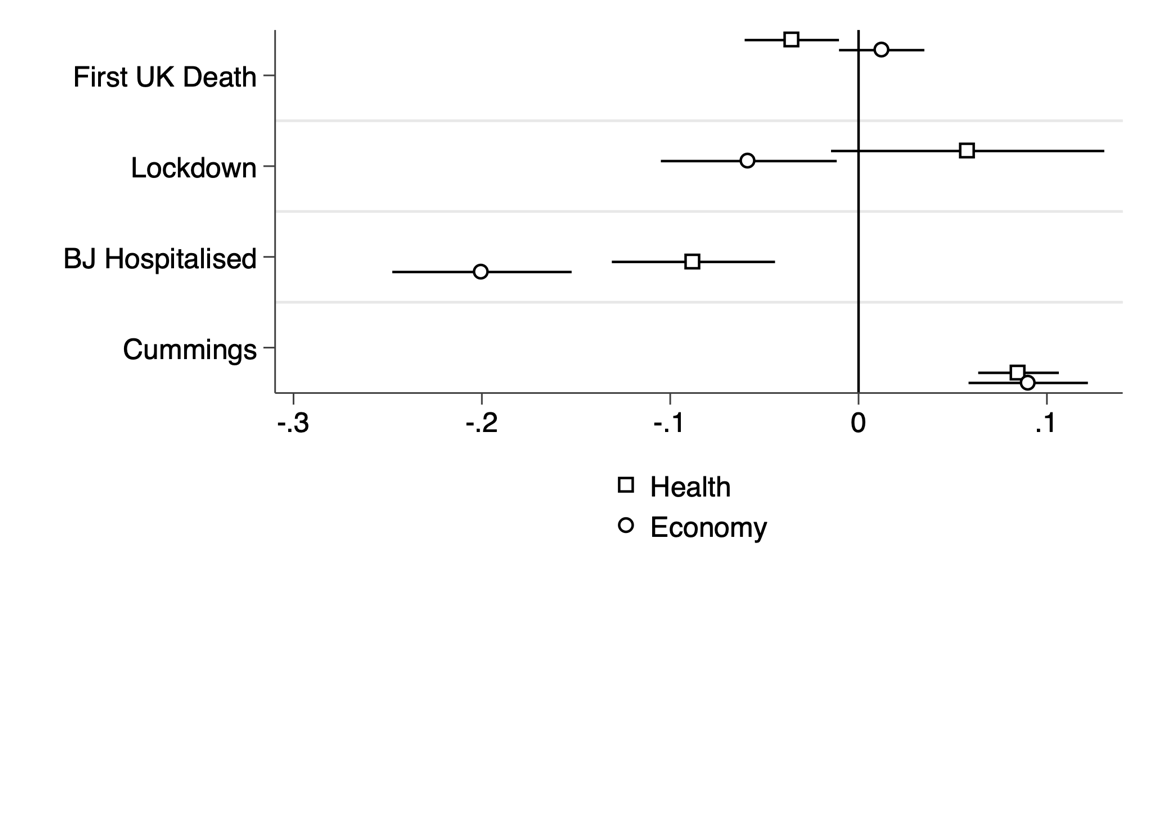


Panel B: England
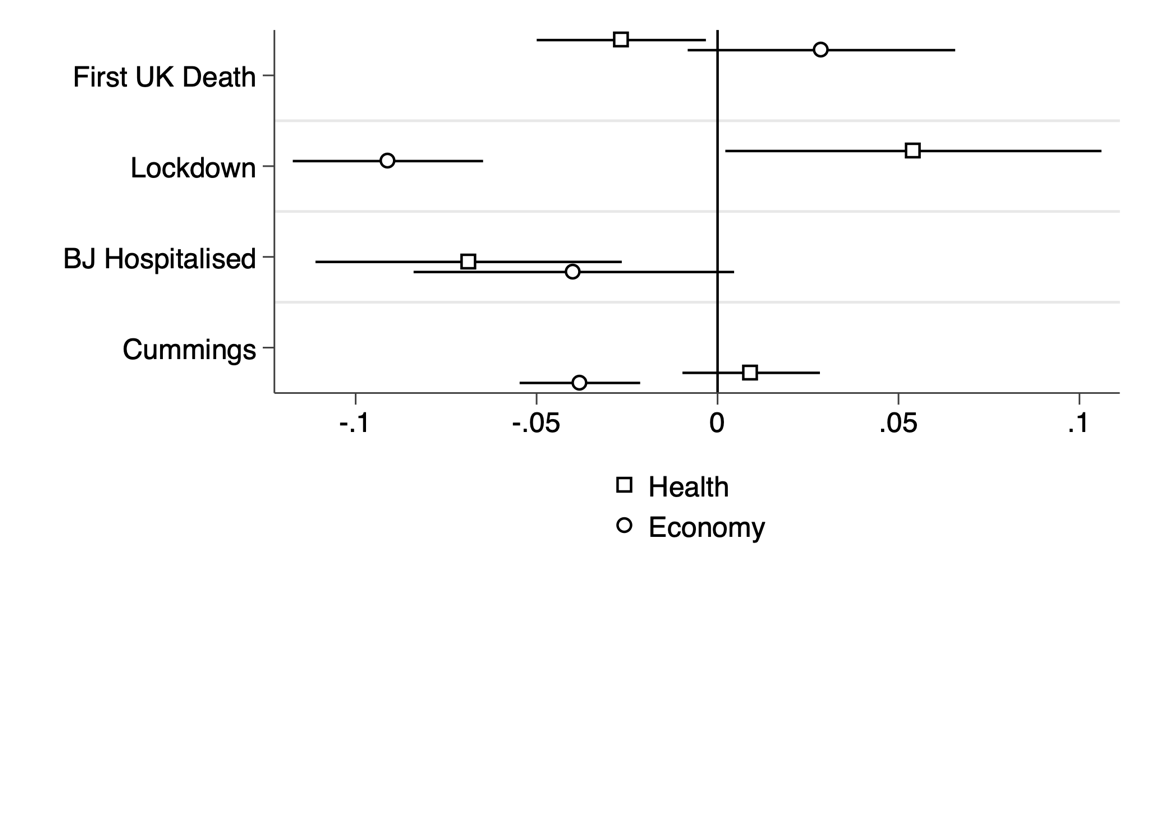


### Figure D3: Impact of partisanship on perceptions of government handling of health and the economy


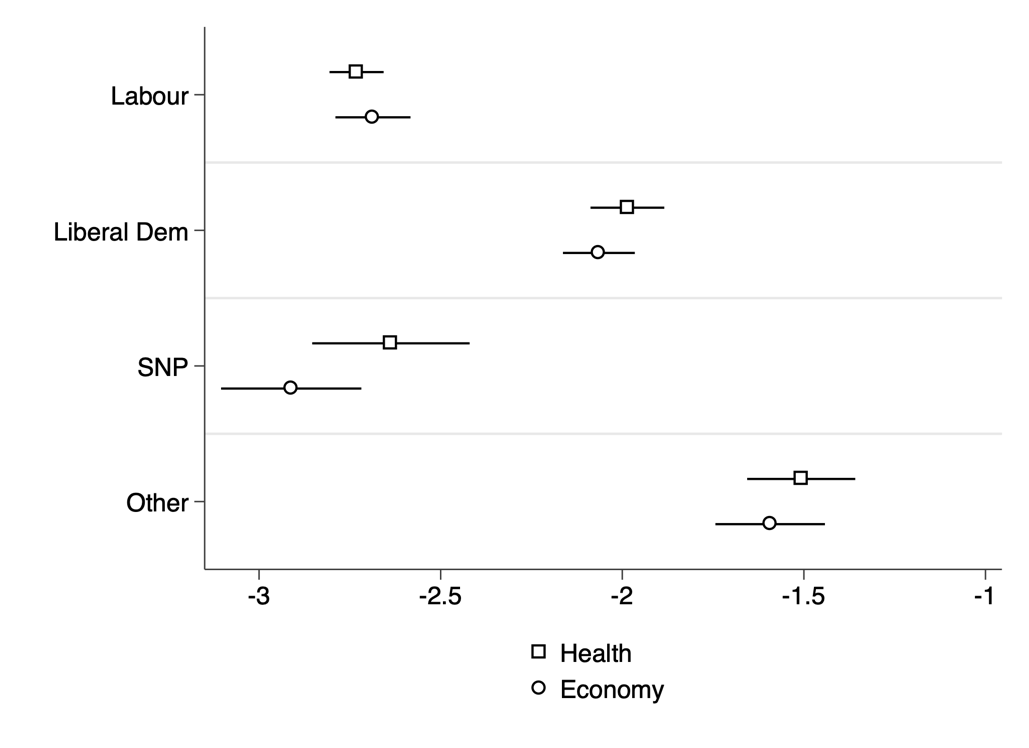


### Figure D4: Average marginal effect of events on government handling of economy, conditional on partisanship, and including basic and full models

| *Panel A: First death*  *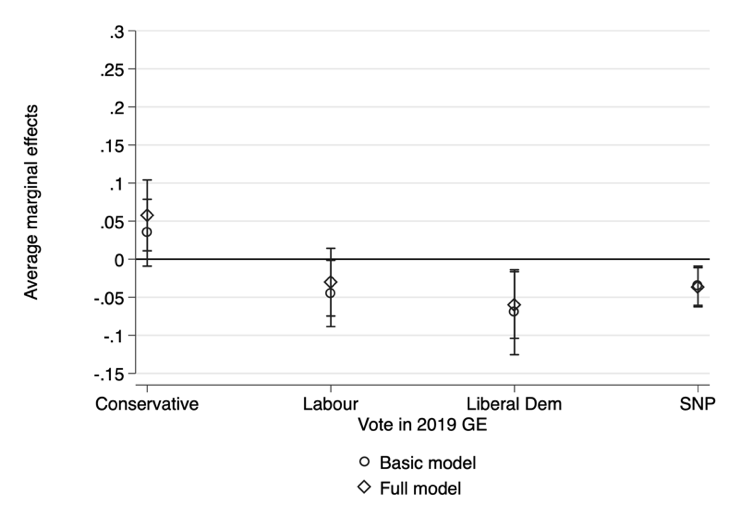* | *Panel B Lockdown*  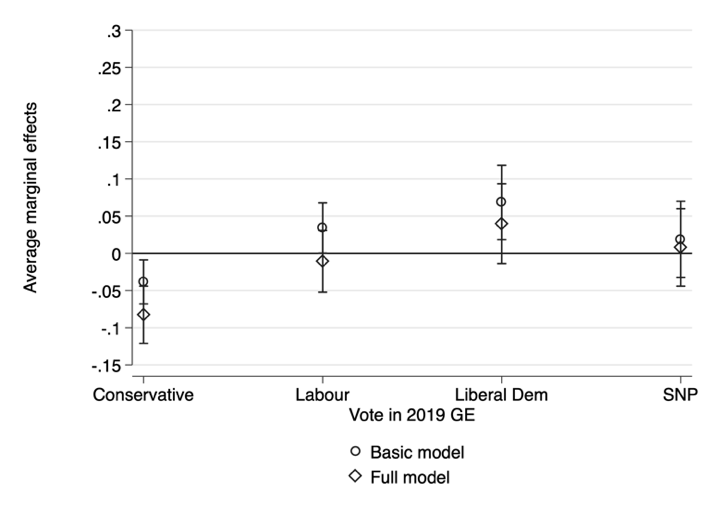 |
| --- | --- |
| *Panel C: Boris Johnson’s hospitalisation*  *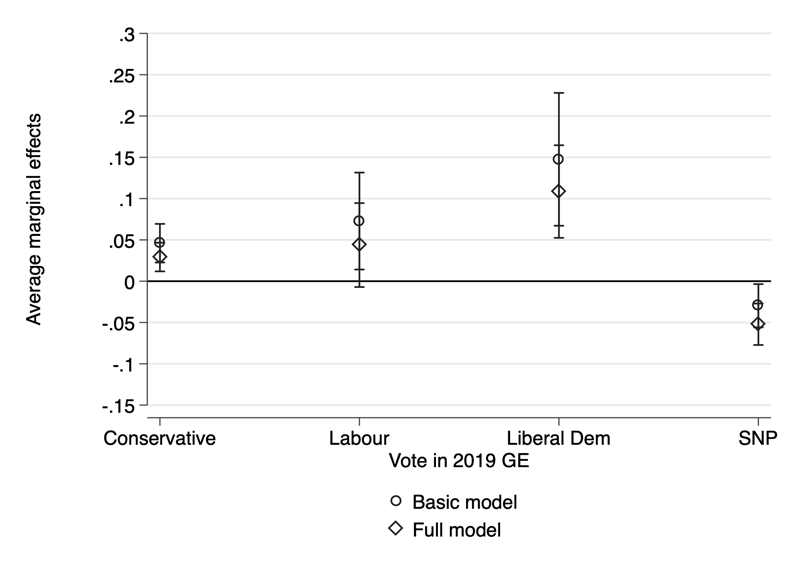* | *Panel D: Cummings’ scandal*  *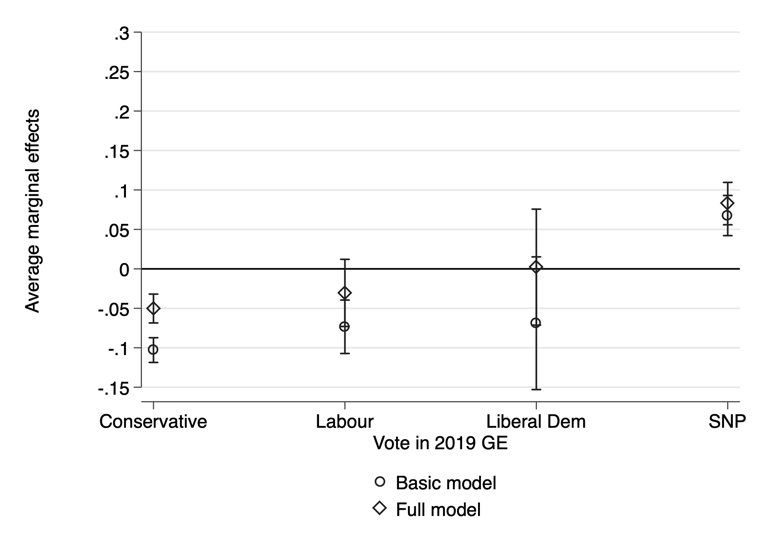* |

*Note: these figures plot average marginal effects of each event conditional on partisan affiliation on the dependent variable. The “Other” category was omitted from the graphs but was present in the regression specification.*

### Figure D5: Average marginal effect of events on government handling of health, conditional on partisanship, and including basic and full models

| *Panel A: First death*  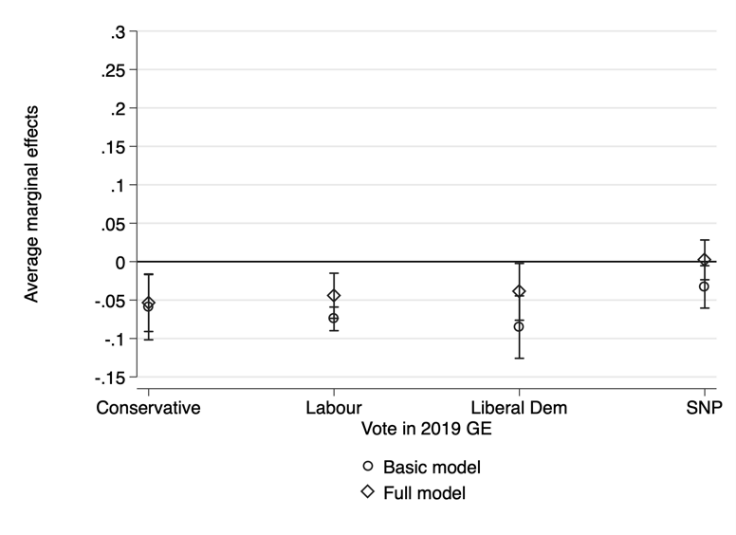 | *Panel B: Lockdown*  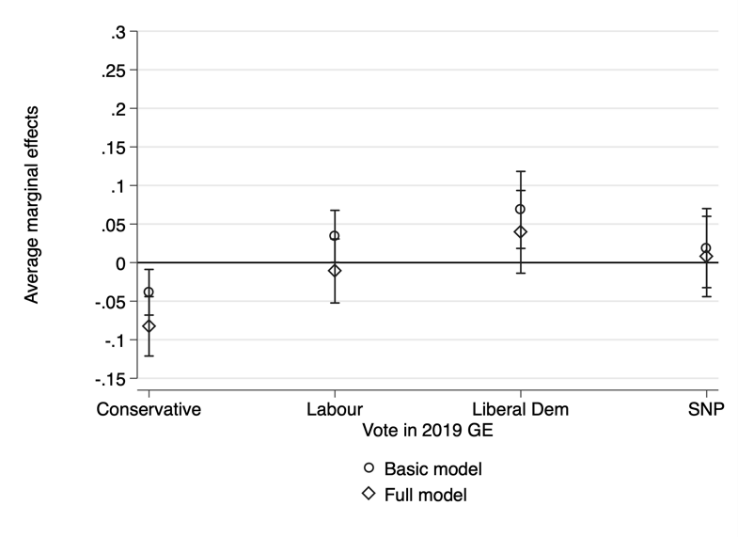 |
| --- | --- |
| *Panel C: Boris Johnson’s hospitalisation* 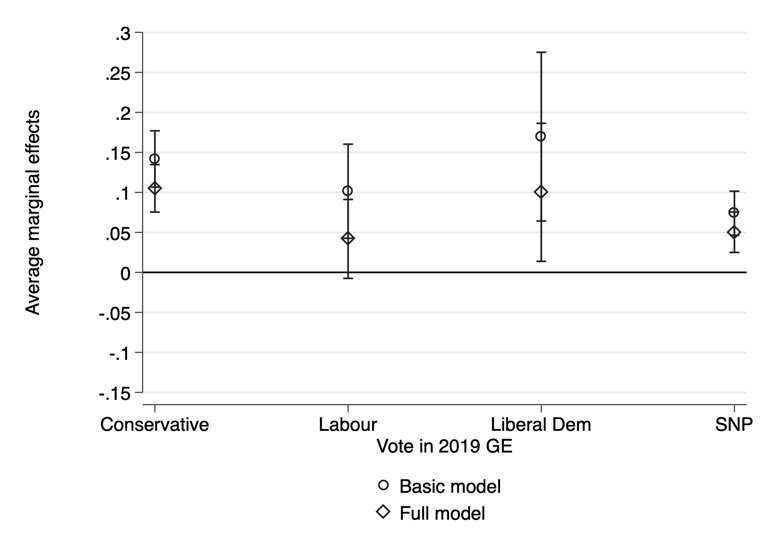 | *Panel D: Cummings’ scandal*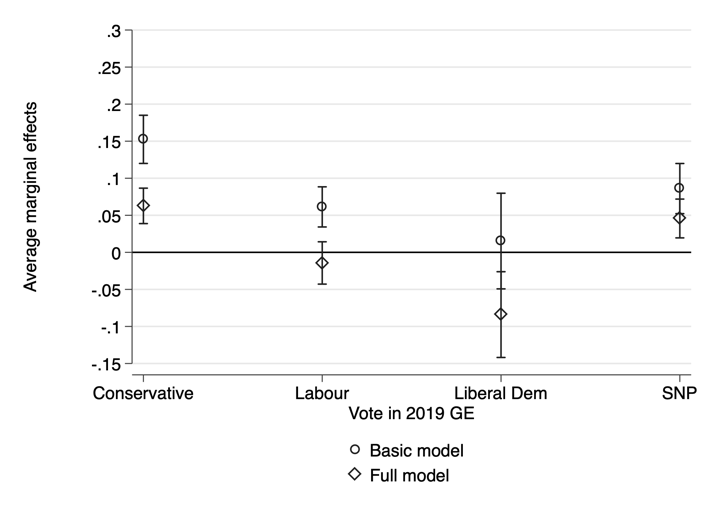 |

*Note: these figures plot average marginal effects of each event conditional on partisan affiliation on the dependent variable. The “Other” category was omitted from the graphs but was present in the regression specification.*

### Figure D6: Average marginal effect of events on government handling of economy, conditional on partisanship. Logistic models.

| *Panel A: First death*  *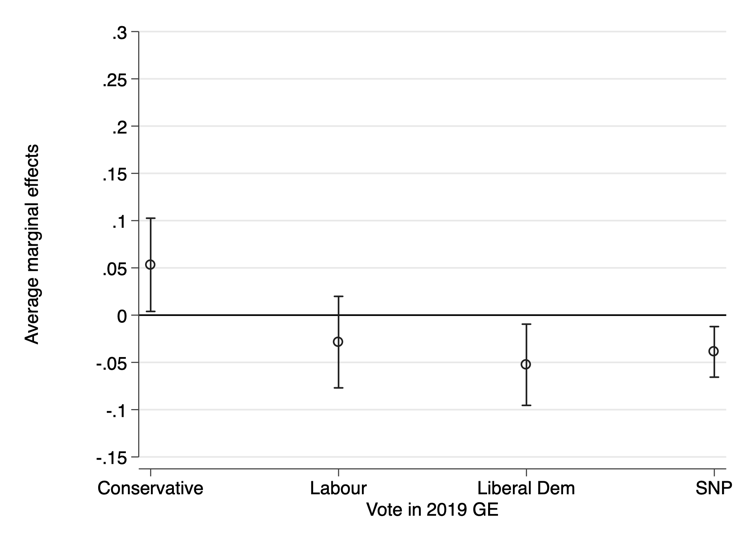* | *Panel B Lockdown*  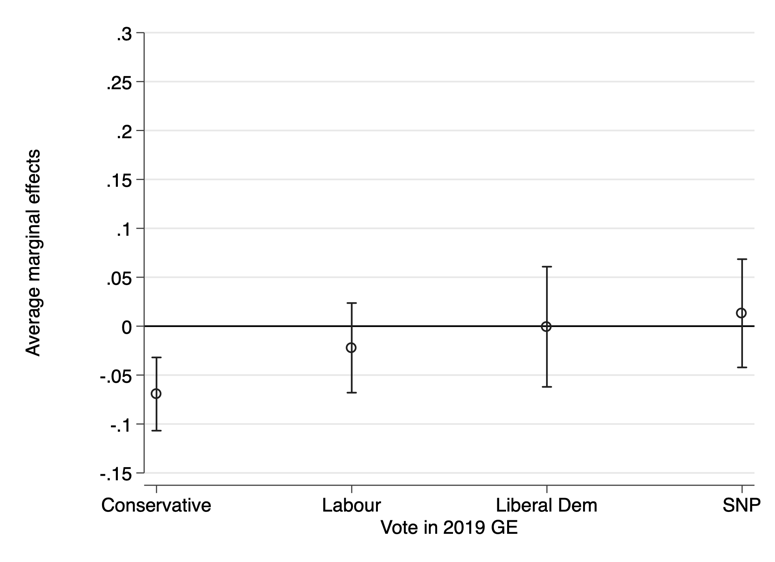 |
| --- | --- |
| *Panel C: Boris Johnson’s hospitalisation*  *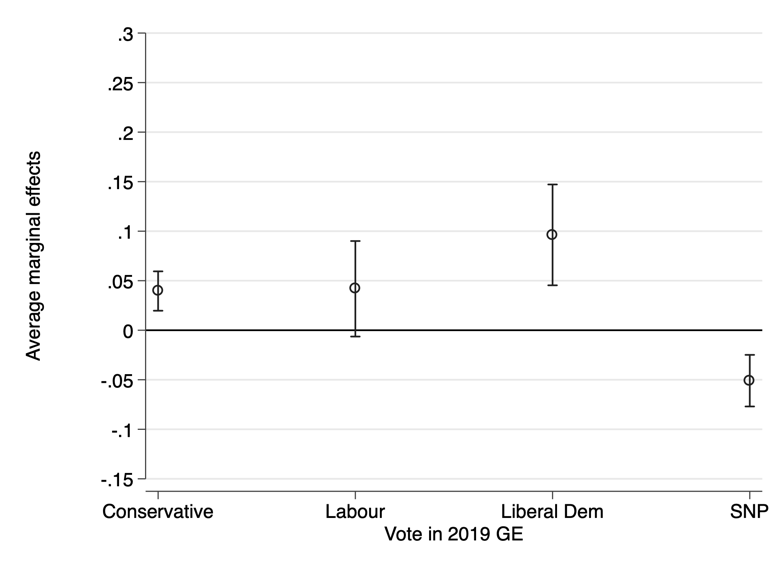* | *Panel D: Cummings’ scandal*  *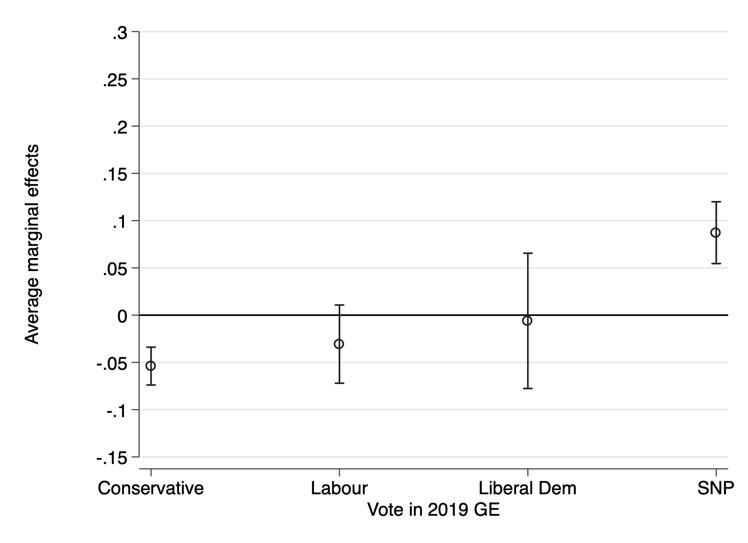* |

*Note: these figures plot average marginal effects of each event conditional on partisan affiliation on the dependent variable. The “Other” category was omitted from the graphs but was present in the regression specification.*

### Figure D7: Average marginal effect of events on government handling of health, conditional on partisanship. Logistic models.

| *Panel A: First death*  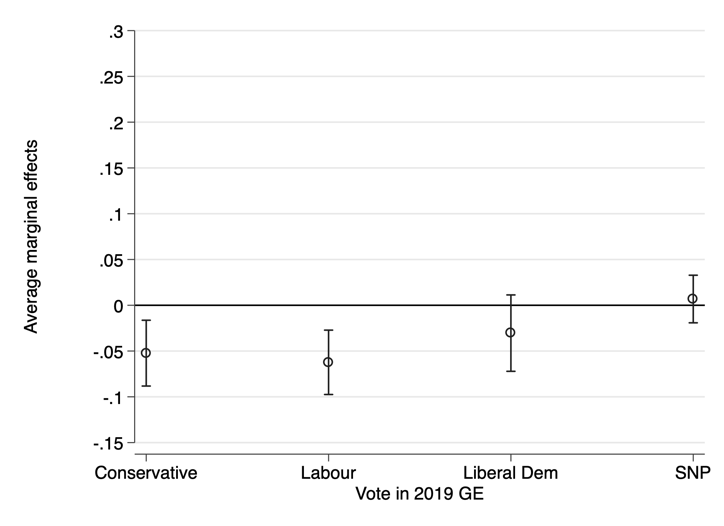 | *Panel B: Lockdown*  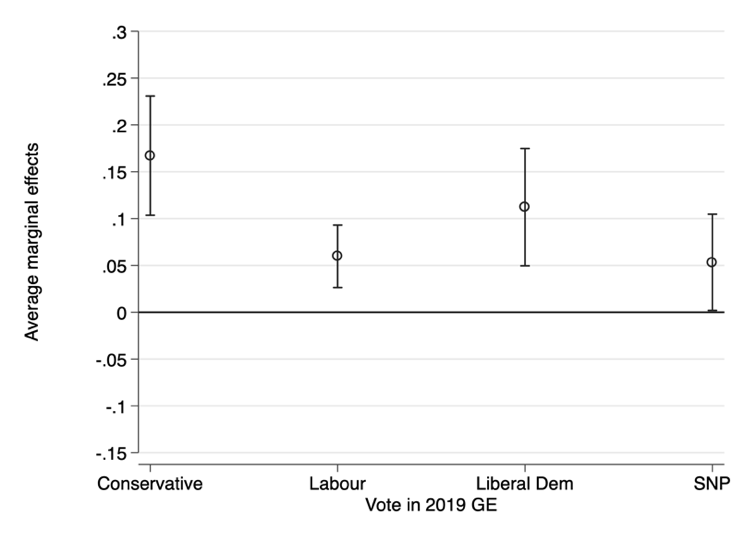 |
| --- | --- |
| *Panel C: Boris Johnson’s hospitalisation*  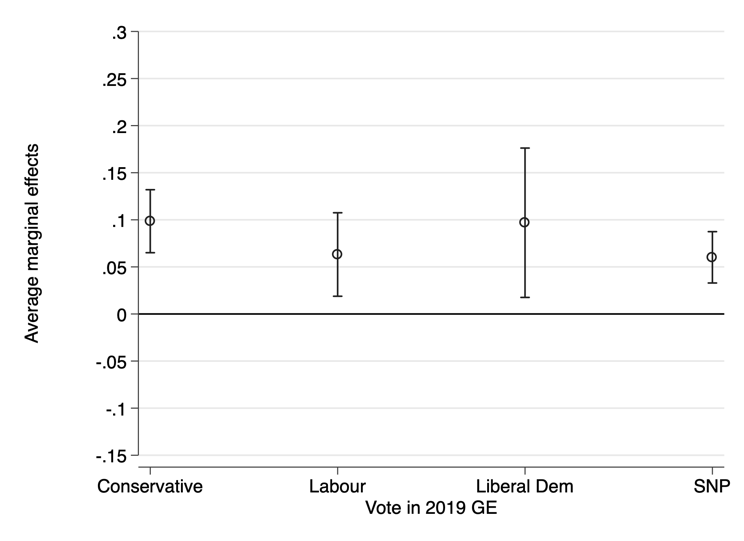 | *Panel D: Cummings’ scandal*  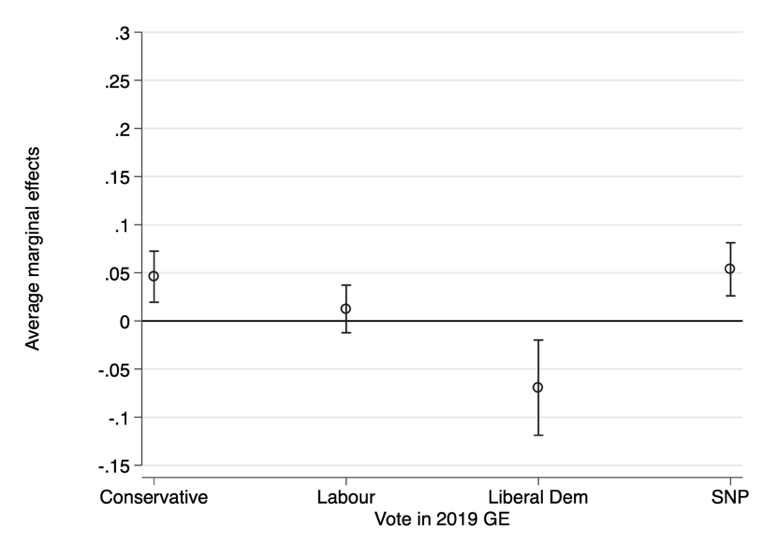 |

*Note: these figures plot average marginal effects of each event conditional on partisan affiliation on the dependent variable. The “Other” category was omitted from the graphs but was present in the regression specification.*

### Figure D8: Average marginal effect of Cummings’ scandal on government handling of economy, conditional on partisanship and excluding June.


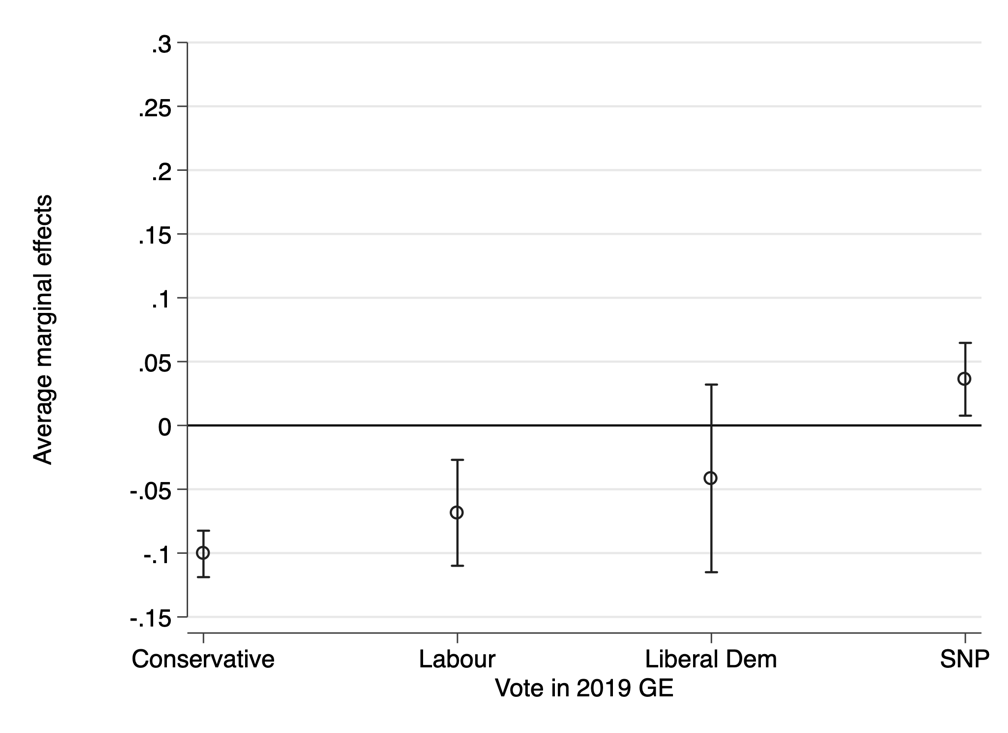


*Note: the “Other” category was omitted from the graphs but was present in the regression specification.*

### Figure D9: Average marginal effect of Cummings’ scandal on government handling of health, conditional on partisanship and excluding June.


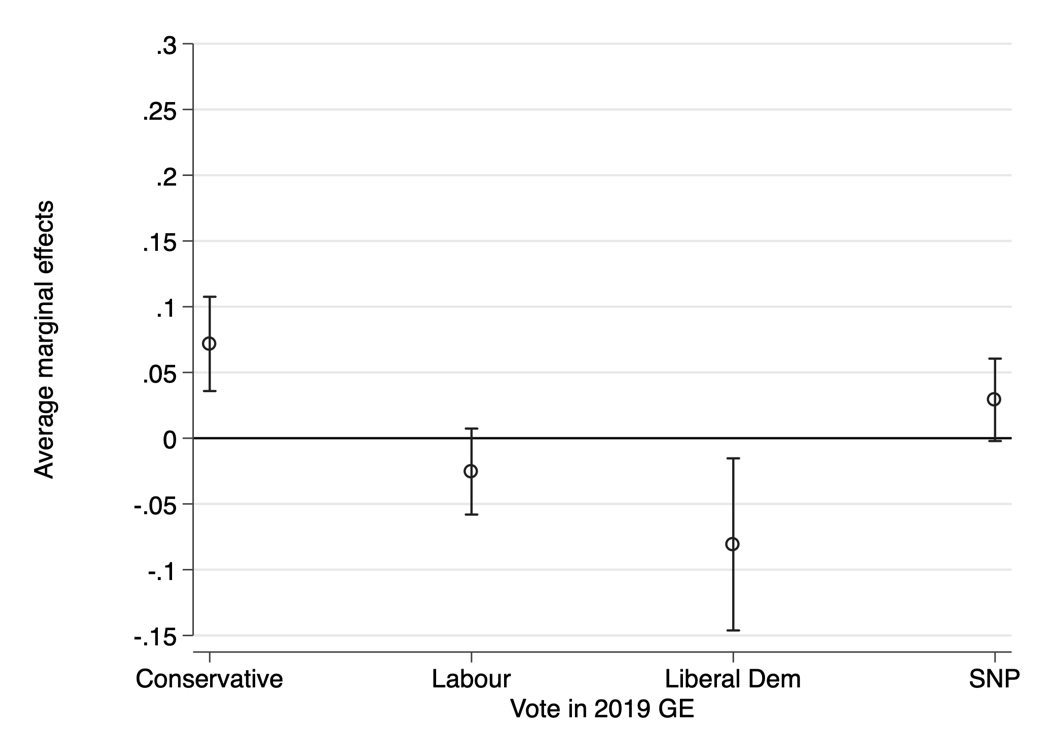


*Note: the “Other” category was omitted from the graphs but was present in the regression specification.*

### Figure D10: Average marginal effect of Cummings’ scandal on government handling of economy, conditional on partisanship and controlling for hospitalization.


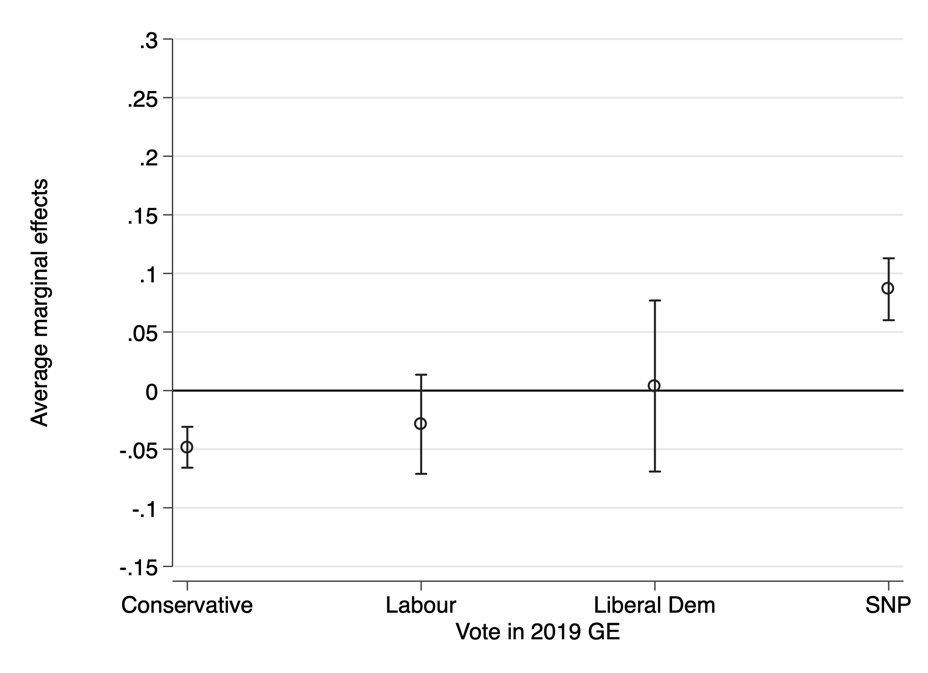


*Note: the “Other” category was omitted from the graphs but was present in the regression specification.*

### Figure D11: Average marginal effect of Cummings’ scandal on government handling of health, conditional on partisanship and controlling for hospitalization.


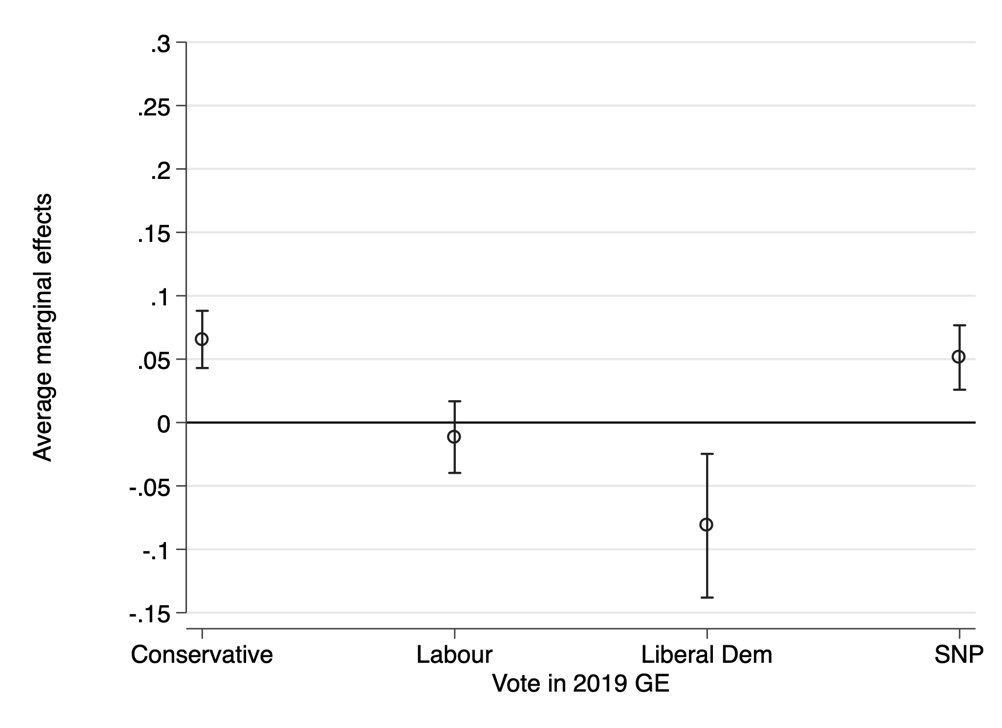


*Note: the “Other” category was omitted from the graphs but was present in the regression specification.*

### Figure D12: Average marginal effect of events on government handling of economy, conditional on partisanship and without controlling for Covid-19 related death

| *Panel A: First death*  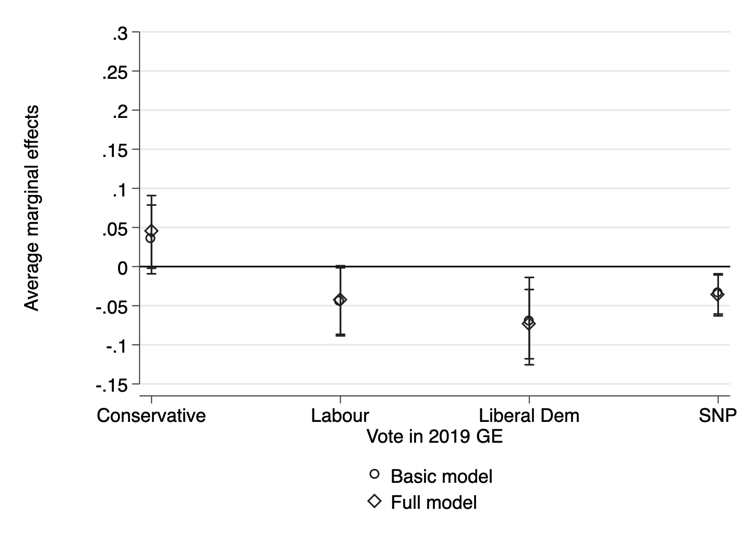 | *Panel B: Lockdown*  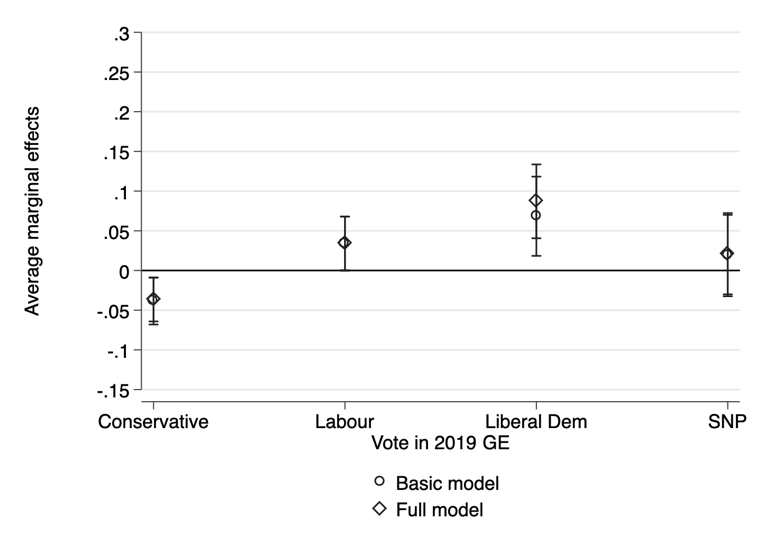 |
| --- | --- |
| *Panel C: Boris Johnson’s hospitalisation*  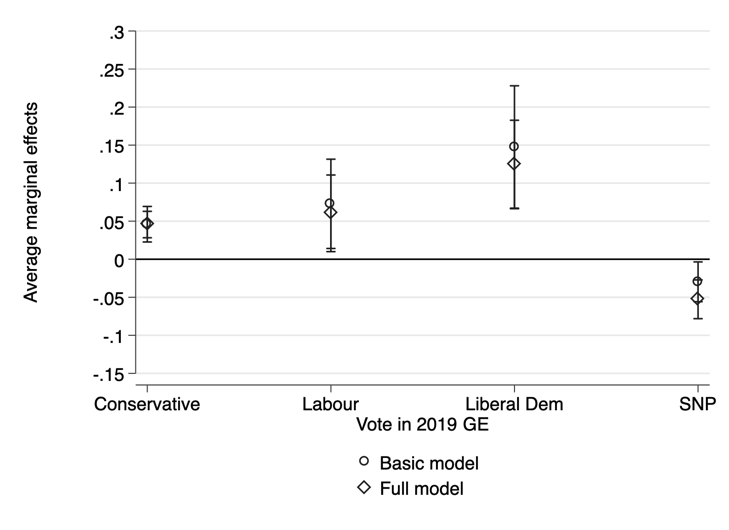 | *Panel D: Cummings’ scandal*  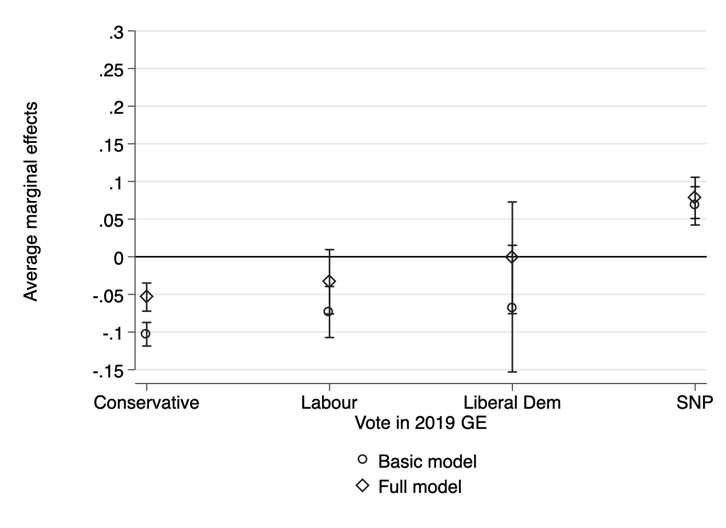 |

*Note: these figures plot average marginal effects of each event conditional on partisan affiliation on the dependent variable. The “Other” category was omitted from the graphs but was present in the regression specification.*

### Figure D13: Average marginal effect of events on government handling of health, conditional on partisanship and without controlling for Covid-19 related death

| *Panel A: First death*  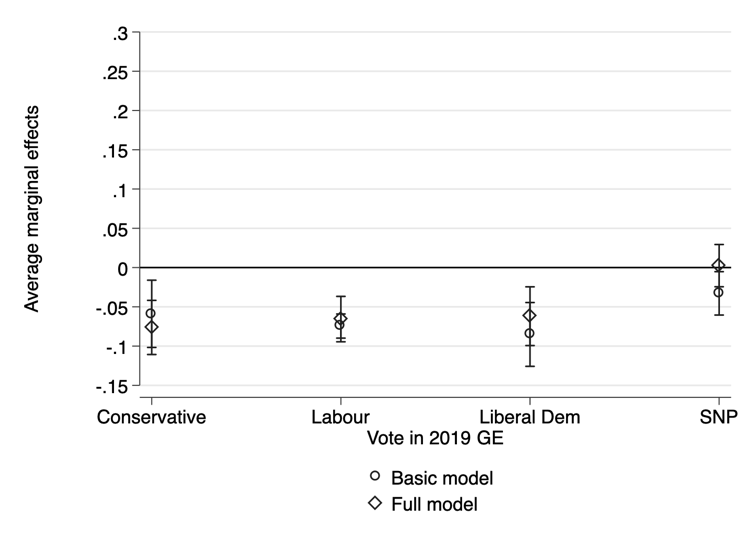 | *Panel B: Lockdown*  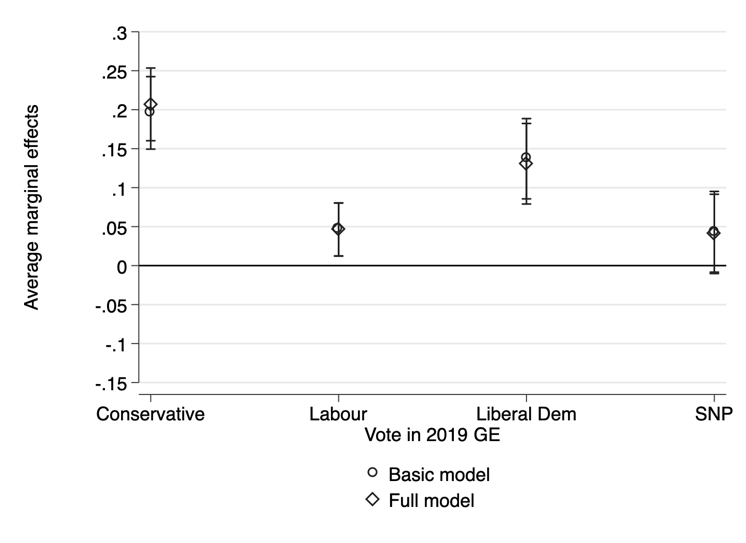 |
| --- | --- |
| *Panel C: Boris Johnson’s hospitalisation*  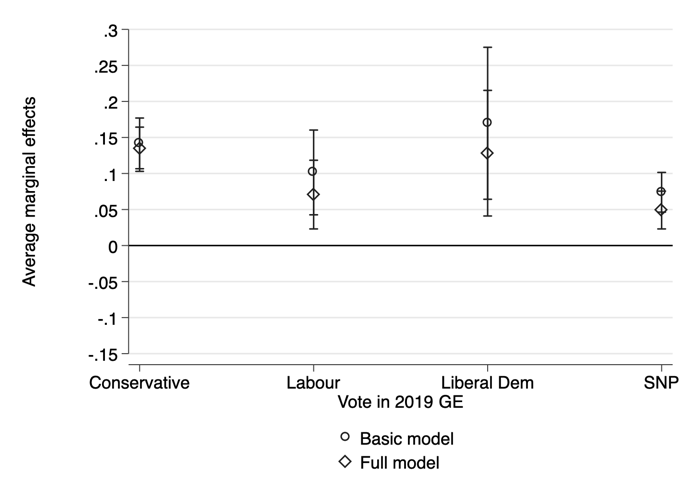 | *Panel D: Cummings’ scandal*  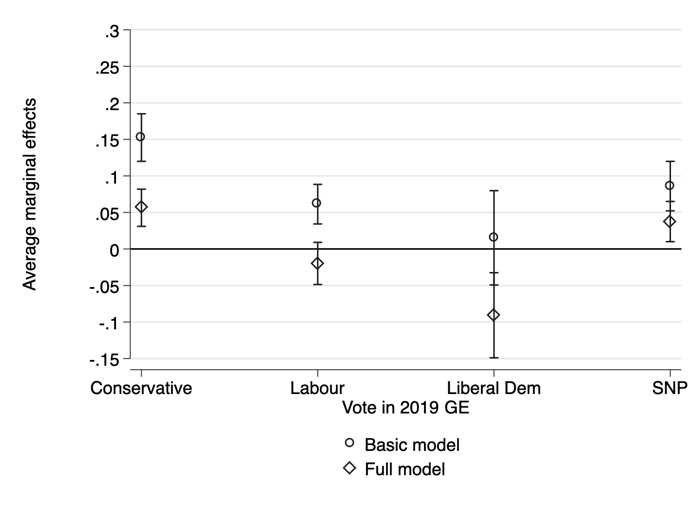 |

*Note: these figures plot average marginal effects of each event conditional on partisan affiliation on the dependent variable. The “Other” category was omitted from the graphs but was present in the regression specification.*

### Table D1: Marginal effects of events on government handling of economy, conditional on partisanship for SNP voters

| **Marginal effects (right) Event (below)** | **Effect for SNP voters for whole sample** | **Effect for SNP voters for Scottish sample** |
| --- | --- | --- |
| *First death* | -0.037 | -0.016 |
|  | [-0.063; -0.011] | [-0.047; 0.014] |
| *Lockdown* | 0.008 | -0.068 |
|  | [-0.044; 0.060] | [-0.176; 0.041] |
| *Johnson’s hospitalisation* | -0.052 | -0.071 |
|  | [-0.077; -0.027] | [-0.091; -0.052] |
| *Cummings* | 0.083 | 0.067 |
|  | [0.056; 0.110] | [0.037; 0.097] |

*Note: 95% confidence intervals are shown in brackets below the estimate for the effect of being an SNP voter. We present results for the whole sample and then rerun our analysis while restricting the sample to Scotland. The results appear relatively consistent across both samples. The only exception concerns the first death which was negatively and statistically significantly associated with perceptions of government’s competence for SNP voters in the full sample but not in the Scottish subsample.*

## Predictive marginal effects for economy.

### Table D2. Predictive probability of respondent stating that government is handling the economy well.

| **Event (right)** | **First death** | | **Lockdown** | | **Johnson hospitalization** | | **Cummings scandal** | |
| --- | --- | --- | --- | --- | --- | --- | --- | --- |
| Party (below) | *Before* | *After* | *Before* | *After* | *Before* | *After* | *Before* | *After* |
| *Conservatives* | 0.829 | 0.882 | 0.862 | 0.793 | 0.830 | 0.869 | 0.833 | 0.779 |
|  | [ 0.818; 0.841] | [ 0.835; 0.930] | [ 0.848; 0.877] | [ 0.766; 0.820] | [ 0.818; 0.841] | [ 0.852; 0.886] | [ 0.821; 0.844] | [ 0.764; 0.794] |
| *Labour* | 0.266 | 0.237 | 0.278 | 0.256 | 0.263 | 0.305 | 0.266 | 0.236 |
|  | [ 0.254; 0.278] | [ 0.190; 0.284] | [ 0.246; 0.310] | [ 0.236; 0.276] | [ 0.251; 0.276] | [ 0.259; 0.352] | [ 0.254; 0.278] | [ 0.196; 0.275] |
| *Conservative-Labour* | 0.563 | 0.645 | 0.584 | 0.537 | 0.567 | 0.564 | 0.567 | 0.543 |
| *Liberals* | 0.400 | 0.347 | 0.397 | 0.397 | 0.395 | 0.491 | 0.398 | 0.392 |
|  | [ 0.380; 0.419] | [ 0.309; 0.385] | [ 0.359; 0.436] | [ 0.363; 0.430] | [ 0.375; 0.414] | [ 0.443; 0.539] | [ 0.379; 0.418] | [ 0.323; 0.461] |
| *SNP* | 0.225 | 0.186 | 0.217 | 0.230 | 0.226 | 0.175 | 0.221 | 0.309 |
|  | [ 0.194; 0.257] | [ 0.161; 0.212] | [ 0.173; 0.260] | [ 0.193; 0.266] | [ 0.194; 0.257] | [ 0.151; 0.199] | [ 0.189; 0.254] | [ 0.271; 0.346] |
| *Mean* | 0.430 | 0.413 | 0.439 | 0.419 | 0.428 | 0.460 | 0.430 | 0.429 |
| SD | 0.276 | 0.320 | 0.292 | 0.260 | 0.277 | 0.302 | 0.279 | 0.242 |
| T-test stat signf | ** | ** | ** | ** | ** | ** | ** | ** |

*Note: 95% confidence intervals are shown in brackets. The last row indicates the statistical significant difference between the predicted probabilities of different voter groups for each column, itself capturing the predicted probability of respondent stating that government is handling economy well before and after our four events. This test statistic takes into account multiple hypothesis testing using the Benjamini and Hochberg FDR correction but the results are not affected when we also apply a Bonferroni adjustment.*

### Table D3. Predictive probability of respondent stating that government is handling the health well.

| **Event (right)** | **First death** | | **Lockdown** | | **Johnson hospitalization** | | **Cummings scandal** | |
| --- | --- | --- | --- | --- | --- | --- | --- | --- |
| Party (below) | *Before* | *After* | *Before* | *After* | *Before* | *After* | *Before* | *After* |
| *Conservatives* | 0.673 | 0.621 | 0.585 | 0.753 | 0.668 | 0.767 | 0.67 | 0.716 |
|  | [ 0.659; 0.687] | [ 0.589; 0.653] | [0.544; 0.627] | [0.726; 0.780] | [0.655; 0.682] | [ 0.736; 0.798] | [ 0.656; 0.684] | [ 0.694; 0.738] |
| *Labour* | 0.139 | 0.077 | 0.1 | 0.16 | 0.134 | 0.198 | 0.137 | 0.149 |
|  | [ 0.129; 0.149] | [ 0.043; 0.110 ] | [0.077; 0.123] | [0.145; 0.175] | [ 0.125; 0.144] | [ 0.154; 0.241] | [ 0.127; 0.147] | [ 0.126; 0.172] |
| *Conservative-Labour* | 0.534 | 0.544 | 0.485 | 0.593 | 0.534 | 0.569 | 0.533 | 0.567 |
| *Liberals* | 0.246 | 0.216 | 0.178 | 0.291 | 0.242 | 0.338 | 0.248 | 0.179 |
|  | [ 0.225; 0.267] | [ 0.180; 0.251] | [0.137; 0.220] | [0.259; 0.323] | [ 0.221; 0.262] | [ 0.261; 0.415] | [ 0.228; 0.269] | [ 0.134; 0.224] |
| *SNP* | 0.148 | 0.155 | 0.113 | 0.167 | 0.146 | 0.206 | 0.146 | 0.2 |
|  | [0.121; 0.175] | [ 0.135; 0.174] | [0.079 ; 0.148] | [0.129; 0.205] | [0.119; 0.172] | [ 0.182; 0.230] | [ 0.119; 0.173] | [ 0.176; 0.224] |
| *Mean* | 0.302 | 0.267 | 0.244 | 0.342 | 0.298 | 0.377 | 0.3 | 0.311 |
| SD | 0.252 | 0.243 | 0.23 | 0.28 | 0.252 | 0.268 | 0.251 | 0.271 |
| T-test stat signf | ** | ** | ** | ** | ** | ** | ** | ** |

*Note: the last row indicates the statistical significant difference between the predicted probabilities of different voter groups for each column, itself capturing the predicted probability of respondent stating that government is handling NHS well before and after our four events. This test statistic takes into account multiple hypothesis testing using the Benjamini and Hochberg FDR correction but the results are not affected when we also apply a Bonferroni adjustment*
